# Multiple‐Layer Chitosan‐Based Patches Medicated With LTX‐109 Antimicrobial Peptide for Modulated Local Therapy in the Management of Chronic Wounds

**DOI:** 10.1002/mabi.202400375

**Published:** 2024-10-14

**Authors:** Sara Bernardoni, Lucia Ferrazzano, Chiara Palladino, Chiara Artusi, Francesca Bonvicini, Elisabetta Campodoni, Giovanna Angela Gentilomi, Alessandra Tolomelli, Monica Sandri

**Affiliations:** ^1^ Institute of Science Technology and Sustainability for Ceramics (ISSMC) National Research Council (CNR) Via Granarolo 64 Faenza 48018 Italy; ^2^ Department of Chemistry “Giacomo Ciamician” Alma Mater Studiorum – University of Bologna Via Selmi 2 Bologna 40126 Italy; ^3^ Department of Pharmacy and Biotechnology Alma Mater Studiorum – University of Bologna Via Massarenti 9 Bologna 40138 Italy; ^4^ Microbiology Unit IRCCS Azienda Ospedaliero‐Universitaria di Bologna Via Massarenti 9 Bologna 40138 Italy

**Keywords:** antimicrobial peptides, biocompatible materials, chronic wounds, local therapy, medicated patches, tissue regeneration

## Abstract

In response to the critical issue of chronic wound management, this research explores the development of a multiple‐layer biomaterial loaded with LTX‐109 a novel broad‐spectrum topical antimicrobial peptide currently investigated for the treatment of bacterial skin infections. The novel patch is conceived to load and preserve the function of LTX‐109, release it on site in a progressive manner, and therefore make available a device for simultaneous wounds disinfection and tissues healing. Chitosan, tannic acid and glycerol along with the solvent casting process are selected for the development of a multilayer structure in which each single layer is designed by choosing a specific composition and stability to tune its behavior and function. On the top, a protective layer to protect the wound from external contaminations, in the middle a medicated layer loaded with LTX‐109 and at the bottom a multifunctional layer to modulate the release of LTX‐109. Extensive characterizations show that the patch meets the essential requirements for creating an effective wound healing environment, such as absorption of exudate, maintenance of good oxygen and moisture permeability, biodegradability, biocompatibility, and sustained release of LTX‐109 with fully retained antibacterial activity as demonstrated by MIC values obtained against reference bacteria.

## Graphical abstract

This work describes the development of a three‐layer biomaterial patch loaded with the antimicrobial peptide LTX‐109, specifically designed for the disinfection of chronic wounds. The multilayerstructure, made of chitosan (chit), tannic acid (tan) and glycerol (gly), ensures localized and controlled release of LTX‐109, while protecting its functionality from the proteolytic environment, providing a multifunctional approach to stimulate wound healing.

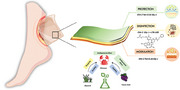

## 1 Introduction

Skin, representing ≈15% of the human body, is the largest organ with the function of protecting against damage and microbial invasion, maintaining the levels of body fluid and electrolytes, facilitating water transfer and CO_2_ exchange as well as the maintenance of body homeostasis and thermoregulation.^[^ ^1^, ^2^ ^]^ When the healthy structure of the skin is disrupted by trauma, burns, surgery, cancer or chronic diseases, microorganisms and pathogens can invade the inner tissues, leading to infections, inflammation, difficult‐to‐heal wounds, thus generally affecting the patient's health and quality of life.^[^ ^1^, ^2^, ^3^ ^]^ Particularly, chronic wounds do not progress toward the normal healing process, are usually associated with severe bacterial infections, and depend on the pathological degree and the health status of the host, and on the environment for resolution.^[^ ^4^ ^]^ If left untreated, these infections give rise to microcolonies that, sheltered by the insurgence of biofilms, alter antibiotics sensitivity and extend to the environment surrounding the lesion, hindering the healing process. Therefore, they greatly impact the healthcare system due to their increasing prevalence and cost. It has been estimated that in developed countries ≈1–2% of the population suffers of chronic wounds, with a similar prevalence rate of heart failures,^[^ ^5^ ^]^ while diabetic foot ulcer, a common complication in patients affected by diabetes mellitus which generally evolves through chronicity, has a comparable 5‐year mortality rate to cancer (31%). These data have remained almost unchanged since 2007, proving the need for new technologies and solutions readily available soon^[^ ^6^ ^]^

Given the increasing social and economic burden that chronic wounds represent, the development of new‐generation dressings devices is a prominent topic. An ideal dressing patch should i) maintain balanced hydration levels through absorption of wound exudates, ii) allow oxygen and moisture exchange across the wound site, iii) provide protection against bacterial infection, iv) enhance epidermal migration, v) promote angiogenesis and connective tissue synthesis, vi) be sterile, non‐toxic, non‐allergic, flexible, non‐adherent and easy to remove without pain for the patient, or alternatively fully bioresorbable, preventing the onset of medical adhesive related skin injuries.^[^ ^7^, ^8^, ^9^ ^]^

In the last decade, research focused mainly on biopolymers due to their excellent biocompatibility, bioresorbability, and ability to mimic the biological and physicochemical features of the native extracellular matrix, which is the architectural support for tissues and plays a pivotal role in the wound healing process^[^ ^10^, ^11^ ^]^ Advanced biopolymer‐based products such as alginates, polyurethanes in foams, hydrogels, hydrocolloids, hydrofibers are already available on the market.^[^ ^12^, ^13^, ^14^, ^15^, ^16^, ^17^ ^]^ They can provide optimal wound moisture levels, prevent tissue maceration by removing excess exudates, emulate body environment conditions, enhance cell growth and differentiation, avoid contact with external bacteria and offer improved biocompatibility compared to traditional dressing.^[^ ^18^ ^]^

Another perk of biopolymers is their potential for careful selection and blending, allowing modulation of their chemical and physical properties to develop composite devices with enhanced characteristics. In particular, in the tissue‐engineering field, multi‐layered patches are emerging as promising alternatives. They offer greater versatility than single‐layer dressings, and the possibility to tune the architecture and the composition of the device to effectively address all the pre‐existing hurdles associated with deep wound care. In that regard, examples of multi‐layer biomaterials for wound dressing applications can be found in literature, obtained by either changing the fabrication method^[^ ^19^ ^]^ or the composition^[^ ^20^ ^]^ of each layer.

However, biomaterials per se do not possess the necessary qualities to fight deep‐seated or chronic infections, therefore, in trying to overcome this limit, advanced dressings containing antimicrobial agents like micronized silver or graphene oxide have recently been put on the market with the aim to protect the wound and to restrain and fight infection.^[^ ^21^, ^22^, ^23^ ^]^

With this work we have developed and investigated an advanced solution to match the ideal characteristics of biopolymers and of innovative antimicrobial peptides by applying a biomimetic approach to the injured tissue, with the aim of recreating an ideal microenvironment for wound disinfection and epidemic tissue regeneration. A three‐layered medicated patch, exhibiting excellent characteristics of biocompatibility and bioresorbability was developed and loaded with the antimicrobial peptide LTX‐109. It is a broad‐spectrum and fast‐acting antimicrobial peptide (AMP), developed by Lytix Biopharma, having successfully completed phase I/II studies for nasal decolonisation of MRSA/MSSA bacterial strains and uncomplicated Gram(+) skin infections,^[^ ^24^, ^25^, ^26^ ^]^ whose mechanism of action is believed to involve an initial binding to negatively charged membrane components on the bacterial cell, followed by a prompt membrane disruption and cell lysis.^[^ ^27^ ^]^ Antimicrobial peptides are prevalent in various living organisms like insects, plants, invertebrates, and vertebrates, where they play a pivotal role in natural defence. They exhibit potent antimicrobial activity against a wide spectrum of microorganisms, including Gram‐negative and Gram‐positive bacteria, fungi, and viruses.^[^ ^28^, ^29^, ^30^ ^]^ Consequently, these peptides are highly promising as antimicrobial agents for treating chronic, infected wounds thanks to their low tendency to induce resistance in vitro, which is a crucial feature in the care of this kind of lesions.^[^ ^31^, ^32^, ^33^, ^34^ ^]^ However, their instability and rapid degradation within the wound site by protease still represents a major challenge to be addressed. As a result, topical administration of AMP often proves ineffective, as the active substance is diluted by exudates and inactivate by in situ degradation, thus needing higher dosage and dosing frequencies to achieve the desired therapeutic effect, without considering that repeated applications can be very painful for the patient.^[^ ^35^, ^36^, ^37^ ^]^

To contrast this drawbacks a novel approach is required, and local and sustained release of antimicrobial peptides, through conjugations with biomaterials like chitosan, may represent a valuable alternative to obtain new‐generation wound dressing capable of managing AMP delivery, improving their effectiveness by shielding the peptides from harsh wound environment, reducing their susceptibility to degradation, and mitigate antibiotic resistance insurgence.^[^ ^28^, ^29^, ^38^, ^39^ ^]^

Chitosan was selected as the primary component of the multilayer patch here presented, due to its array of properties, including biodegradability, biocompatibility, bio‐adhesivity, immune‐stimulator capacity and bacteriostatic attributes.^[^ ^40^, ^41^, ^42^ ^]^ Thanks to its cationic nature, the antibacterial activity of chitosan has been widely reported in the literature,^[^ ^43^ ^]^ as well as extensive studies have shown its ability to accelerate wound contraction and healing, both in medical and veterinary domains.^[^ ^44^, ^45^ ^]^

In recent years various chitosan‐ and chitosan‐derived products have emerged in the market. However, films of pure chitosan suffer from brittleness and lack of flexibility. To address this, chitosan is often blended with a plasticizer, molecules like polyols that can decrease the intermolecular attractions between the adjacent polymer chains by interacting with the acetamide groups of chitosan, and in particular glycerol‐ a polyol known for its plasticization efficacy, widespread availability, and biocompatibility‐ emerges as a preferred choice.^[^ ^43^, ^46^ ^]^ Additionally, the mechanical properties of chitosan, as well as its toughness and strength can be enhanced through cross‐linking. Among different options of chemical cross‐linkers tannic acid,^[^ ^47^, ^48^ ^]^ a natural plant‐based polyphenol compound serves as a suitable cross‐linker for chitosan/glycerol films. It readily interacts with biopolymers like collagen, chitosan, albumin, and gelatin through non‐covalent interactions like H_2_‐bonding and hydrophobic effects, not increasing the toxicity of the material,^[^ ^48^ ^]^ other than providing several biological functions such as antiviral, anti‐inflammatory, antioxidant, and antimicrobial properties, which made it the therapy of choice for the clinical treatment of burns in the mid‐1920s.^[^ ^49^, ^50^ ^]^

The purpose of this work is to develop and investigate a three‐layer chitosan‐based device, conveniently cross‐linked and loaded with the selected antimicrobial peptide LTX‐109, capable of ensuring the drug preservation, its local and controlled release, and exert a sustained antibacterial therapy, which is essential for promoting wound healing and skin tissue regeneration. Specifically, it has been designed to consist of a highly cross‐linked outer skin‐like layer that is durable and acts as a physical barrier to protect the wound from bacterial invasion from the external environment, while allowing gas and moisture to pass through, an intermediate non‐cross‐linked layer loaded with the antimicrobial peptide LTX‐109 to ensure preserved activity and controlled drug release directly to the wound site, and a lower layer with a low degree of crosslinking to regulate the local release of LTX‐109 and to protect the antimicrobial peptide from proteolytic enzymes commonly found in the wound environment. Extensive characterization, including swelling ratio, water vapor transmission rate, degradation rate, drug release kinetic, antimicrobial activity, and biocompatibility were performed to evaluate whether the patch meets the essential requirements for an effective wounds treatment, as well as its ability to load, preserve, and deliver a medication effective for the healing of infected wounds.

## 2 Experimental Section

### 2.1 Reagents

Low molecular weight chitosan (CS, MW 50 000—190 000 Da, based on viscosity; Deacetylation degree > 75%; viscosity 20–300 cps), tannic acid (TA, MW 1701.20 g mol^−1^), glycerol (Gly, MW 92.09 g mol^−1^, density 1.25 g ml^−1^), phosphate buffer saline (PBS) were supplied by Sigma‐Aldrich (MO, USA). Common high‐purity chemical reagents were purchased from Sigma‐Aldrich. Ultrapure water (0.22 mS, 25 °C) was used for the synthesis.

All materials, solvents and reagents for the synthesis of LTX‐109 were obtained from commercial suppliers and used without further purification. The solvents used were of high‐performance liquid chromatography (HPLC) reagent grade. In particular, the Fmoc amino acids and resins were supplied by Iris Biotech, Merck or Fluorochem. The coupling reagents were purchased from Merck or Novabiochem. Piperidine was supplied by Merck. Dimethylformamide (DMF), other organic solvents (dicholorometane (DCM), *N, N*‐Diisopropylethylamine (DIPEA), Trifluoroacetic acid (TFA) and HPLC‐grade acetonitrile (CH_3_CN) were purchased from Merck. Deuterated solvents for NMR analysis (dimethylsulfoxid d6‐DMSO; methanol d4‐MeOH) were purchased from Eurisotop.

### 2.2 Patches Preparation

#### 2.2.1 Single‐Layer Patches (SL‐Patches)

Chitosan solution (2% w/w) was prepared by adding 2 g of chitosan powder in 100 g of acetic acid aqueous solutions (1%, v/v) containing 1%w/w of glycerol, as plasticizer, and variable amounts of tannic acid (0, 0.04, 0.16% w/w), as chemical cross‐linker. The limpid polymeric solutions thus obtained and composed as summarized in **Table** **1**, were mechanically stirred for 2 h, then were sonicated with ultrasound for 30 min to eliminate bubbles formed during the stirring. To obtain a thin membrane 60 g of each solution were poured into a Petri dish of *Ø *= 90 mm and placed at room temperature in a fume hood for 48 h until the complete solvent evaporation.

**Table 1:** Patches code and chemical composition.

Code	Solution composition	Patch composition	Position in the multi‐layer
SL_1_‐patch (Chit2Tan0.16Gly1)	Chitosan (2%w/w) Tannic acid (0.16%w/w) Glycerol (1%w/w)	Chitosan 63.3w% Tannic acid 5.1w% Glycerol 31.6w%	Top‐layer (skin‐type protective layer)
SL_2_‐patch (Chit2Gly1)	Chitosan (2%w/w) Glycerol (1%w/w)	Chitosan 66.7w% Glycerol 33.3w%	Middle‐layer (layer for medication)
SL_3_‐patch (Chit2Tan0.04Gly1)	Chitosan(2%w/w) Tannic acid (0.04% w/w) Glycerol (1%w/w)	Chitosan 65.8w% Tannic acid 32.9w% Glycerol 1.3w%	Bottom‐layer (regenerative layer)

#### 2.2.2 Multi‐Layer Patch (ML‐Patch)

A three‐layer structure was obtained by using the same polymeric solutions prepared as described in Section 2.2.1 and by solvent casting and layer‐by‐layer methods. Twenty grams of the first solution with composition SL_1_‐patch (Table 1), with the highest content of tannic acid (0.16% w/w), was poured into a Petri dish of *Ø* = 90 mm and dried in an oven at 40 °C for ≈4 h until a partially‐dry and still adhesive surface was obtained. Twenty grams of the second solution with composition SL_2_‐patch (Table 1), without tannic acid, were then spread on top of the first layer and dried in the same conditions. Finally, 20 g of the third solution with composition SL_3_‐patch (Table 1), with a low content of tannic acid (0.04% w/w), were added on top of the partially dried bilayer, and the three‐layer biomaterial was placed, at room temperature, in a fume hood for 48 h until complete solvent evaporation.

### 2.3 LTX‐109 Synthesis

The solid‐phase synthesis of LTX‐109 (AMP) was carried out manually or using a CSBio‐CS136X peptide synthesizer (automated syntheses).

The manual synthesis of LTX‐109 was performed using a standard protocol for SPPS. Briefly, the peptide sequence was assembled in a glass syringe fitted with a porous polyethylene disc, connected to a vacuum source to remove excess reagents and solvents. PS‐TrtCl resin (loading 1.03 mmol g^−1^, 0.750 g) was swelled in 3.5 ml of DCM for 30 min at room temperature. The loading of the first amino acid, N‐Fmoc‐Arg(Pbf)‐OH (1.5 eq), was performed in DCM (0.3 m): to a solution of amino acid in DCM, DIPEA (3 eq) was added and the mixture stirred at room temperature for 5 min. The solution was loaded onto the resin and gently stirred for 1 h. The resin was filtered off and washed with 2 × 4.5 ml DCM. Capping of unreacted functionalities of the resin was performed using, first, a solution of DCM (2 ml), MeOH (90 µl) and DIPEA (400 µl), stirring the resin for 15 min. After filtering the resin and washing with 3 × 4.5 ml of DCM, a second capping solution was added, mixing DCM (2.3 ml) and acetic anhydride (210 µl) and stirring the resin for 15 min. The resin was filtered off and washed with 3 × 4.5 ml DCM and 3 × 3.5 ml DMF. The deprotection of the N‐terminal functionality of the growing peptide was performed using 20% piperidine/DMF solution (2 × 3.5 ml × 15 min). After this step, the resin was washed with 3 × 3.5 ml of DMF. The following couplings were performed using DIC/OxymaPure (1:1) as coupling reagents for the activation of N‐protected amino acids. In particular, N‐Fmoc‐Tbt‐OH (2 eq) and N‐Boc‐Arg(Pbf)‐OH (3 eq) were sequentially introduced in 1:1 ratio with coupling reagents: the amino acid was dissolved in DMF (0.4 m) with OxymaPure; after stirring at room temperature for 5 min, DIC was added and the mixture stirred for further 5 min. The solution was loaded onto the resin, which was gently stirred at room temperature for 1 h. The resin was filtered off and washed with DMF (3 × 3.5 ml). When elongation of the sequence was completed, the resin was finally washed with 3 × 3.5 ml of DCM before the peptide was cleaved from the solid support. The cleavage cocktail, made of 1% TFA (1 eq, 150 µl) in DCM (15 ml), was added portion‐wise (5 × 3 ml × 5 min), keeping controlled the temperature to avoid over warming the resin. The solution was filtered and poured into a solution of 12.6% pyridine (189 µl) in MeOH (2.7 ml). After the removal of solvents under vacuum, the crude COOH/NHBoc peptide was obtained. It was then coupled with 2‐phenethylamine (1 eq), using DIC (1.5 eq) and OxymaPure (1.5 eq) in DCM (0.1 M) as coupling reagents. After monitoring of reaction completeness by TLC analysis, the solution was washed with H_2_O (5 ml), HCl 0.1 m (5 ml), NaHCO_3_, _sat. sol_. (5 ml), brine (5 ml). The organic phase was anidrifyed by Na_2_SO_4_ and dried under vacuum to afford the crude protected peptide. It was finally deprotected with a solution of TFA:TIPS:DDT:H_2_O = 92.5:2.5:2.5:2.5 (3 ml). After drying under vacuum, the fully deprotected peptide LTX‐109 was obtained and purified by RP‐HPLC, affording LTX‐109 with purity >99.5%. Automated syntheses of LTX‐109 were performed at room temperature using the same conditions reported for manual protocol.

#### 2.3.1 Purification and Characterization of LTX‐109

RP‐HPLC purification of LTX‐109 was performed on an Agilent 1260 Infinity II using the following setup:

Column: Daisogel‐SP‐120‐Ods‐BIO C18 250 × 4.6 mm 10 µm;

Temperature: 25 °C; 

Detector: UV 224 nm;

Gradient: H_2_O + 0.08%TFA (mobile phase A) and CH_3_CN + 0.08%TFA (mobile phase B) (**Table** **2**).

**Table 2:** Conditions used for LTX‐109 RP‐HPLC.

Column volumes	Title flow rate [ml min^−1^]	A [%]	B [%]
2	2	70	30
5	2	/	/
6.7	1	70	30
1	1	20	80
4	2	10	90

HPLC‐MS analysis were performed on an Agilent 1260 Infinity II system coupled to an electrospray ionization mass spectrometer (positive‐ion mode, m/z = 100–3000 amu, fragmentor 30 V), using ChemStation software for data processing. The analytical method adopted for peptide characterization was setup as follows:

Column: Phenomenex Luna C18 5 µm, 250 × 4.6 mm;

Temperature: 25 °C;

Injection volume: 10 µL;

Detector: UV 220 nm;

Flow: 1.0 mL min^−1^

Gradient: H_2_O + 0.08%TFA (mobile phase A) and CH_3_CN + 0.08%TFA (mobile phase B) (**Table** **3**).

**Table 3:** Gradient parameters for HPLC‐MS analysis.

Time	A [%]	B [%]
0	80	20
15	40	60
20	10	90
25	10	90
30	80	20

The NMR spectra were recorded using an INOVA 400 MHz instrument with a 5 mm probe. The sample was dissolved in d_4_‐MeOH. All chemical shifts were quoted relative to the deuterated solvent signals. The chromatogram of LTX‐109 and NMR signals map, demonstrating the purity and the chemical structure of the AMP were reported in the Figure S1 (Supporting Information).

### 2.4 Antimicrobial Peptide LTX‐109 Loading in SL_2_‐Patch and ML‐Patch

Antimicrobial peptide (AMP) loading was performed by adding the dissolved LTX‐109 directly into the polymeric solution, before solvent evaporation and layer formation. Fifteen milligrams of the selected antimicrobial peptide LTX‐109 were solubilized in 100 µl of ethanol at room temperature and added to the solution of chitosan and glycerol (SL_2_‐patch composition, Table 1) at a ratio of 0.06% w/w ad homogenized by magnetic stirring achieving a limpid solution with the composition reported in **Table** **4**. Then the SL_2_‐patch‐L and the AMP‐loaded ML‐patch (ML‐patch‐L) were prepared as described above.

**Table 4:** Code and chemical composition of the loaded patch (SL_2_‐patch‐L).

Code	Solution composition	Patch composition	Position in the multi‐layer
SL_2_‐patch‐L (Chit2Gly1LTX)	Chitosan (2%w/w) Glycerol (1%w/w) LTX‐109 (0.06%w/w)	Chitosan 65.4w% Glycerol 32.6w% LTX‐109 2 w%	Middle‐layer (AMP‐loaded layer)

### 2.5 Characterization of the Developed Single and Multi‐Layer Samples

#### 2.5.1 Morphological Characterization

Patches morphology was evaluated by environmental scanning microscopy (ESEM, Quanta 200 FEG, FEI Company, Hillsboro, OR, USA). The samples were prepared by fixing them onto aluminum stubs using carbon tape and observed without coating in a low vacuum setting.

The macroporosity of the patches was evaluated using the water‐squeezing method, which measured the amount of water inside a sample before and after sample squeezing. The method was based on the principle that water was present in small and big pores inside the polymer network. The water in its latter form was relatively free and represented the porosity requirement for cell penetration and proliferation. The method measured the macropores volume percentage with the following procedure: the scaffold was equilibrated in MilliQ water for two hours and weighed (M_swollen_); subsequently, it was squeezed to remove the water filling the pores and weighed again (M_squeezed_). Macropores volume was calculated using Equation (1):

(1) $$\begin{array}{ccc} \mathrm{Macropore}\;\mathrm{Volume}\;\mathrm{percentage}\left(\%\right) & = & \left(\left(M_{\mathrm{swollen}}-M_{\mathrm{squeezed}}\right)\right)\\ & & /M_{\mathrm{swollen}})\times 100 \end{array}$$

The values were expressed as the mean ± standard error (*n* = 3).^[^ ^51^ ^]^

#### 2.5.2 Physical Characterization

##### Swelling Behavior

The fluid uptake ability of the patch was monitored by the water sorption study under physiological conditions. Cylindrical samples of the layers (diameter 6 mm and height 0.2 mm) were immersed in PBS solution at 37 °C until the scaffolds reached a swelling saturation. At regular time intervals (0.5, 1, 2, 4, 6, 24, and 48 h) the sample was withdrawn, and the excess water was removed using filter paper. After measuring the weight, the equilibrium swelling ratio was calculated using Equation (2):

(2) $$\begin{array}{c} \mathrm{Swelling}\;\mathrm{ratio}=\left(\mathrm{wet}\;\mathrm{weight}-\mathrm{dry}\mathrm{weight}\right)/\left(\mathrm{wet}\;\mathrm{weight}\right)\times 100 \end{array}$$

The measure was repeated in triplicate on different samples.^[^ ^52^ ^]^

##### In Vitro Degradation

For the degradation tests, cylindrical samples of the patch (diameter 6 mm and height 0.2 mm) were freeze‐dried for two days to remove excess water present in the structure; then they were immersed in phosphate buffered saline (PBS, pH 7.2) in the presence of 0.1% (wt/vol) NaN_3_ at 37 °C. At specific time intervals, (1, 3, 7, and 14 days) the samples were taken out from the medium and washed twice with Milli‐Q water. The samples were freeze‐dried again for two days and subsequently weighed. The degradation percentage (D (%)) was evaluated using the Equation (3):

(3) $$D\left(\%\right)=\left(W_{i}-W_{f}\right)/W_{i}\times 100$$

where W_i_ is the initial weight of the freeze‐dried sample before immersion in PBS and *W*_f_ is the weight of the freeze‐dried sample at a specific time point.^[^ ^52^ ^]^

##### Water Vapor Transmission Rate (WVTR)

To evaluate the moisture permeability of the prepared patch, their water vapor transmission (WVTR) was measured by the ASTM E96 standard method.^[^ ^53^ ^]^ First, a vial (diameter = 1.50 cm) was prepared, covered with the prepared patch and weighed (*W*_0_). The volume of water in the cup was 10 mL. An open vial and a closed vial with their lid were considered positive and negative controls. After being placed into a humidity chamber kept at 37 °C and 50% relative humidity for 24, 48, and 72 h, they were weighed again (*W*_t_) and the WVTR of the patch was calculated using Equation (4):

(4) $$\mathrm{WVTR}\left(gm^{\left(-2\right)}d^{\left(-1\right)}\right)=\left(\left(W_{t}-W_{0}\right)/t\right)/A$$

where *A* is the exposed area of the patch (m^−2^), and t is the time of measurement (day). The values were expressed as the mean ± standard error (*n *= 3).

##### Infrared Spectroscopic Analyses (FTIR‐ATR)

Fourier transform infrared spectra of the samples were measured with a Nicolet 5700 spectrometer (Thermo Fisher Scientific Inc., Waltham, MA, USA) in ATR mode (FTIR‐ATR) using ATR iD7accessory. Instrumental resolution was set up to 4 cm^−1^ and 16 scans were collected per sample in the range spanning from 4000 to 400 cm^−1^.

##### Mechanical Properties

The tensile strength, the elongation at break and the Young modulus of the dry patches were measured on a screw‐driven load frame for mechanical testing at room temperature (Zwick‐Roell Z050). The patches were cut into quadrangles of 50 mm × 8 mm and the samples were held between two clips at 50 mm apart. The extension rate was 5 mm min^−1^. The ultimate tensile strength (UTS) and the elongation at break (EB %) were obtained from the stress–strain curves, using the Equations (5)–(6) reported below, while the Young modulus was calculated as the slope of the linear part of the stress–strain curve (strain range 2–13%).

(5) $$\mathrm{UTS}\left(N/mm^{2}\right)=F_{\mathrm{break}}/A$$

(6) $$\mathrm{EB}\left(\%\right)=L/L_{0}\times 100$$

where *F* = breaking load, *A* = initial cross‐sectional area of the sample, *L*_0_ = original length of the sample and *L* = increase in length at breaking point. Each patch was tested 10 times.^[^ ^54^ ^]^

### 2.6 Contact Angle Measurement

The wettability of the prepared patches was measured using a contact angle system (Drop Shape Analyzer DSA 30S, Krüss GmbH) at ambient temperature with a 10 µl water droplet. The samples were cut into a 10 mm × 10 mm square and glued to a microscope slide to have a flat surface. Measurements were collected from the sample surface at two different times: initial time (*T*_0_) and after 60 s (*T*_60_) (*n* = 4).

### 2.7 Antimicrobial Peptide Release Study In Vitro

The release profile of AMP LTX‐109 from fabricated patches (SL_2_‐patch‐L) was evaluated by incubating cylindrical samples (diameter: 8 mm; height: 0.2 mm) in 2 ml of PBS (pH 7.4) and placed in a thermostatic incubator shaker at 37 °C. At various time points (30 min, 1 h, 3 h, 5 h, 7 h, 24 h, 48 h, and 72 h), 200 µl of the buffer solution was collected, and 200 µl of fresh PBS solution was added to ensure the uniformity of the release solution. The cumulative concentration of LTX‐109 released into PBS solution was analyzed by HPLC (High Performances Liquid Chromatography, Agilent 1260 Infinity II, USA). Analysis were carried out using Zorbax Eclipse Plus C18 Column (5 µm, 4.6 × 250 mm), mobile phase CH_3_CN and H_2_O + TFA (0.065% v/v) with the gradient reported in **Table** **5**. The flow rate was 1.0 mL min^−1^, the column temperature was 25 °C, and the UV detector was set at 231 nm.

**Table 5:** Mobile phase gradient conditions. *A* = H_2_O + TFA (0.065% v/v), B = CH_3_CN.

Time	A [%]	B [%]
0	80	20
10	40	60
12	10	90
25	10	90
25.01	80	20
30	80	20

### 2.8 In Vitro Assessment of Patches’ Antibacterial Activity

The antibacterial properties of the fabricated patches (SL_2_‐patch‐L) were evaluated in vitro by testing the inhibitory effect of the LTX‐109 after its release into PBS solution at different incubation times. The effectiveness of the inhibition was assessed against two reference bacterial strains, *Staphylococcus aureus* (*S. aureus*; ATCC 25 923) and *Pseudomonas aeruginosa* (*P. aeruginosa*; ATCC 27 853). These reference strains, purchased from the American Type Culture Collection (ATCC), were selected as representative Gram‐positive and Gram‐negative bacterial models, respectively, frequently associated with chronic wound infections.^[^ ^55^ ^]^

For biological experiments, cylindrical samples of SL_2_‐ patches (diameter 6 mm, height 0.2 mm, weight 7 mg) and ML‐patches (diameter 6 mm, height 0.2 mm, weight 15 mg), loaded with the antimicrobial peptide and unloaded as controls, were incubated with 500 µL of PBS (pH 7.2) at 37 °C, and, at various time intervals (1, 24, and 48 h), 200 µl of the liquid solution were collected and replaced with 200 µl of fresh PBS. The samples, containing the LTX‐109 released in the liquid solution, were subsequently assayed by using a well‐established broth microdilution method, in compliance with the international guidance documents [CLSI, EUCAST]. In detail, *S*. *aureus* and *P*. *aeruginosa* were routinely cultured in 5% blood agar at 37 °C and bacterial inocula were prepared in PBS (pH 7.2), adjusted to an Optical Density at 630 nm (OD630nm) of 0.08–0.1, corresponding to 108 CFU (colony forming units) mL^−1^. Then, the suspensions were diluted 1:200 in Mueller–Hinton Broth (MH–Broth) and then incubated with a tenfold dilution of the starting liquid samples, and subsequent serial twofold dilutions. Bacterial growth was spectrophotometrically measured at 630 nm after 24 h of incubation at 37 °C. The same procedure was used to determine the antibacterial activity of the antimicrobial peptide LTX‐109, herein synthesized (see Section 2.2). For biological assays, LTX‐109 was dissolved in 100% DMSO at a concentration of 10 mg mL^−1^ and thereafter assayed in the range of 25–0.19 µg mL^−1^.

### 2.9 In Vitro Cell Viability and Cytotoxicity Assays

HEL 299 cell line (ATCC CCL137) was used as a model system to investigate the overall effect of the fabricated patches on mammalian cells.

The safety profile of the liquid samples containing the released LTX‐109, prepared as above described, was quantitatively evaluated by measuring both cell viability and proliferation (WST‐8 based method) and the lactate dehydrogenase enzyme (LDH) release from damaged plasma membranes.

Briefly, cells were grown in Eagle's Minimum Essential Medium, supplied with 10% FBS (Fetal Bovine Serum), 100 U mL^−1^ penicillin, and 100 µg mL^−1^ streptomycin), at 37 °C and 5% CO_2_. For experiments, cells were seeded into a 96‐well tissue culture plate at a concentration of 104 cells well^−1^ and incubated for 24 h. Following washes with PBS, the cell monolayer was incubated with 100 µL of the liquid samples recovered after different intervals (1, 24, and 48 h) from the SL_2_‐patch and ML‐patch, AMP‐loaded and unloaded, as controls. The same sample dilutions tested in the antibacterial assays were prepared and tested for their cytotoxicity to ascertain a specific inhibitory effect of the biomimetic AMP‐loaded patches.

The cell viability was assessed by a colorimetric WST‐8 method according to the manufacturer's instructions (CCK‐8, Cell Counting Kit‐8, Dojindo Molecular Technologies, Rockville, MD, USA). Briefly, after 48 h of incubation with the different dilutions of each liquid sample, the culture medium was removed from wells, and collected for the LDH Cytotoxicity assay; the monolayer was washed with PBS, and 100 µL of fresh medium containing 10 µL of CCK‐8 solution were added. The OD450nm values were measured following 4 h at 37 °C.

The cytotoxicity was evaluated by using the Cytotoxicity LDH Assay Kit‐WST (Dojindo Molecular Technologies, Rockville, MD, USA), following the manufacturer's instructions. The amount of LDH enzyme released by damaged HEL 299 cells upon treatment with the liquid samples in the different experimental conditions was then spectrophotometrically determined at OD490nm. The measurement of the concentration of the enzyme in cells was one of the major methods to assess cell death.

### 2.10 Biological Data Analysis

The antibacterial activity of the fabricated patches was expressed in terms of MIC (Minimum Inhibitory Concentration) for *S. aureus* and *P. aeruginosa*. Growth percentage values of the reference strains were determined relative to the untreated positive controls (bacterial cells incubated in MH broth). The lowest concentration of samples that inhibited bacterial growth (≥90%) was noted as the MIC value.

Cell viability and proliferation were expressed as percentage values relative to the untreated Hel 299. Cytotoxicity was determined as percentage values relative to both the 100% lysis controls, included in the test, and untreated controls.

All biological assays were carried out in triplicate, and in at least two independent assays testing different patch samples. One‐tailed *t*‐test and One‐way analysis of variance (One‐way ANOVA) were used to compare the antibacterial activity of the LTX‐109 released in PBS solutions in the different experimental conditions.

## 3 Results and Discussion

In this work, single and three‐layered chitosan‐based patches plasticized with glycerol and cross‐linked with tannic acid in variable amounts for each layer (0; 0.04%; 0.16% w/w), were developed to achieve an effective tunable system able to promote, together with wounds protection and skin regeneration, a local antimicrobial treatment due to the controlled and sustained release of the antimicrobial peptide LTX‐109 with high selectivity, reduced cytotoxicity and strong antibacterial activity.^[^ ^28^, ^29^, ^30^ ^]^ First, each single‐layer patch (SL‐patches: SL_1_ Chit2Tan0.04Gly1, SL_2_ Chit2Gly1 and SL_3_ Chit2Tan0.16Gly1) was prepared and physically and morphologically characterized to evaluate their properties in terms of fluids absorption, chemical and mechanical stability, and vapor permeability, and to assess their suitability to be used for the development of a wound dressing device. Then, based on previous evaluations and results, the SL_2_‐patch was chosen to host the LTX‐109 because of its higher and faster fluid exchange capacity with the surrounding environment, and loaded with the antimicrobial peptide (SL_2_‐patch‐L).

Finally, a multi‐layer patch was prepared by combining the three SL‐patches (ML‐patch), loaded with LTX‐109 in the middle layer (ML‐patch‐L) and fully characterized. Release profiles of LTX‐109 from the SL‐patch‐L and ML‐patch‐L was evaluated by high‐performance liquid chromatography (HPLC) under physiological conditions (PBS, 37 °C), and on the same medium, was assessed the cytocompatibility by in vitro test with HEL 299 cell line (ATCC CCL137) and the preservation of the microbial activity by testing the inhibitory effect of the AMP post‐loading and releasing.

### 3.1 SL‐Patch Morphological Characterization

The SL‐patches were obtained through solvent casting technique. Specifically, chitosan, tannic acid and glycerol were homogenously mixed in a 1% acetic acid solution obtaining three different compositions as reported in Table 1. Sixty grams of each solution was cast on a Petri dish (*ø *= 90 mm) to obtain air‐dried patches with a specific and constant thickness of 0.2 mm in the obtained after complete solvent evaporation in a fume hood for 48 h. The patches were nominated as reported in Table 1.

**Figure 1:**
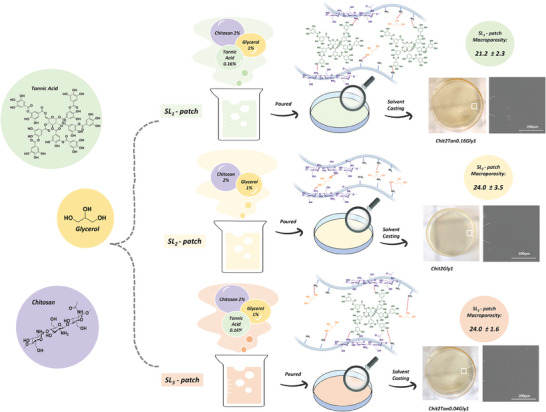
Schematic representation of the passages for the preparation of SL‐patches and their macro and micro‐morphologies. In details are reported the chemical structures of the involved reactive, Tannic Acid, Glycerol, and Chitosan and a hypothetic mechanism of interaction, ESEM images of the superficial morphology of the three patches and their macroporosity evaluated in wet conditions.

The morphological micro‐architecture of the air‐dried patches was evaluated by ESEM and the acquired images reported in **Figure** **1**. As expected, all samples showed a smooth and regular microstructure, without any phase separation, indicating that the process achieved homogenous blends. Since macroporosity is an essential feature to provide cells penetration and proliferation, permeation of nutrients and oxygen, and space for vascularization and tissue growth under in vivo conditions, the percentage of macropores in the assembled patches was measured in wet conditions using the water‐squeezing method.^[^ ^56^ ^]^

All the SL‐patches presented macroporosity ranging between 21% and 24% (insert in Figure 1), with the lower porosity highlighted from the patch containing the higher amount of tannic acid. The result could be related to the ability of tannic acid to cross‐link the chitosan matrix, binding the chains together and creating a more compact structure. However, since there isn't a significant difference between the macroporosity of all samples, this parameter seems to be more conditioned from the fabrication method (solvent casting) rather than patch composition. In fact, as shown by ESEM images, the solvent‐casting method induces the formation of smooth and compact surfaces in all the cases.

**Figure 2:**
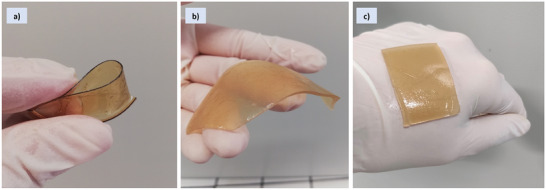
Macroscopic view and behaviour of the SL_1_‐patch in a) dried condition, and b) after soaking/swelling in PBS for 2 h, highlighting the flexibility of the film, both wet and dried, and its adhesiveness when swelled (c).

The same was observed macroscopically (**Figure** **2**a), also highlighting the behaviour of a patch (SL_1_‐patch) before and after swelling in PBS (Figure 2, Video S1, Supporting Information).

### 3.2 Single‐Layer Physico‐Chemical Characterizations

**Figure 3:**
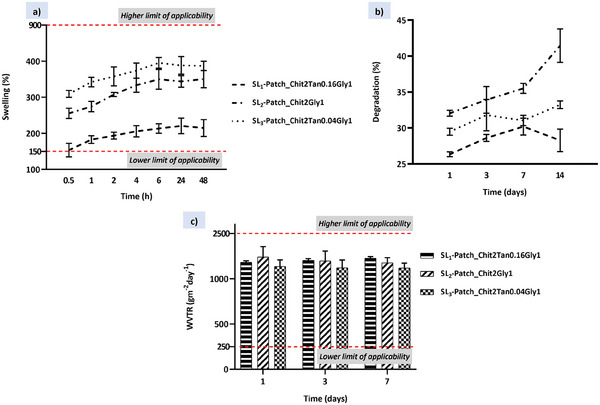
Physical properties of the SL‐patches: a) swelling profiles reporting the water uptake percentages of single patches in PBS solution at 37 °C within 48 h; b) degradation curves. Weight loss percentages of single patches in PBS solution at 37 °C within 14 days; c) water vapour transmission rate of single patches at 1, 3, and 7 days.

Water uptake ability is one of the most important properties to be assessed when developing patches for wound dressing. It correlates with the ability of the material to maintain a considerably moist environment in the wound site, removing surplus wound exudates for healing improvement,^[^ ^57^ ^]^ as well as supporting the appropriate diffusion of nutrients for cells, and managing the bleeding.^[^ ^58^ ^]^ The swelling capacity of SL‐patches was tested in PBS (pH 7.4) at 37 °C and the results are reported in **Figure** **3**a. As shown, the swelling ratio of the patches ranges from 150% to 400%, which is in the range of values for an ideal wound dressing biomaterial (100–900%).^[^ ^9^ ^]^ In particular, the addition of 0.16% (w/w_tot_) of tannic acid into the blend, noticeably reduced the water uptake ability of the patch. This behaviour has already been reported in the literature^[^ ^59^, ^60^, ^61^, ^62^ ^]^ and correlates to the chemical structure of tannic acid, which contains several hydroxyl groups able to establish molecular interactions (hydrogen bonding and Van der Waals interactions) with amine groups in the structure of chitosan, thus blocking the extension of the polymer chains and diminishing their water absorption capacity. In fact, when a lower amount of tannic acid is used (0.04% w/w_t_) the swelling ratio increases proportionally, but if compared with the patch of chitosan and glycerol not cross‐linked (tannic acid = 0%) it is unexpectedly still superior.

This evidence is related to the higher macroporosity of SL_2_‐patch (Chit2Gly1) and SL_3_‐patch (Chit2Tan0.04Gly1), and the intrinsic properties of the material depending on the cross‐linking density and the hydrophilicity, both influencing the swelling process. In fact, the chemistry of tannic acid can effectively influence the cross‐linking of chitosan, as proven by the reduced swelling abilities of the SL_1_‐patch (Chit2Tan0.16Gly1), but at the same time, it is also a strongly hydrophilic molecule that is responsible for drastically increase the number of hydrophilic groups incorporated into the material. Consequently, the increased swelling of the SL_3_‐patch (Chit2Tan0.04Gly1) could be attributed to the contribution in terms of hydrophilicity of tannic acid, which led to the observed results.^[^ ^63^, ^64^ ^]^

Ideally, the degradation rate of bioresorbable patches for wound dressing should match the rate of new tissue formation, thus protecting the infected site from external agents over time.^[^ ^65^ ^]^ Furthermore, the dressing should be stable enough to avoid premature changes, which sometimes disrupt newly formed tissue and cause discomfort to the patient.^[^ ^66^ ^]^ The in vitro degradation of the SL‐patches, Chit2Tan0.04Gly1, Chit2Gly1 and Chit2Tan0.16Gly1 was evaluated in PBS at 37 °C and the results are shown in Figure 3b. Overall, the degradation curves of all the samples, after an initial weight loss that ranges between 26% and 32% during day 1 and depending on the composition of the patches, were almost flat without significant changes, as only a further 2 to 4% of mass loss was registered between day 1 and day 7, which means that the patches have long term stability. The initial weight loss could be attributed to glycerol dissolution in PBS, which is a phenomenon already reported in literature^[^ ^43^, ^67^ ^]^ and confirmed by the fact that the patches at day 14th became brittle after drying. On day 14th, the degradation rate of SL_1_‐patch and SL_3_‐patch containing tannic acid (Chit2Tan0.04Gly1 and Chit2Tan0.16Gly1) remained stable at ≈28–32%, while the degradation rate of SL_2_‐patch (Chit2Gly1) increased at 42%, indicating that the degradation rate decreases as the increase of tannic acid content, and demonstrating that the cross‐linking of the chitosan matrix depends on tannic acid.^[^ ^48^ ^]^ It is worth noting that also the SL_2_‐patch, not cross‐linked with tannic acid, did not completely dissolve in PBS after two weeks, showing that the combination of both the fabrication method (solvent casting) and the addition of glycerol as a plasticizer produced a compact patch and helped in stabilizing its structure. In particular, the stabilizing effect of glycerol on chitosan films was already reported in literature^[^ ^68^ ^]^ and it could be attributed to the interaction between chitosan and glycerol. As demonstrated by Domjan et al.^[^ ^69^ ^]^ by performing solid‐state NMR spectroscopy on plasticized chitosan films, the glycerol molecule is able to form H‐bonds with the glucosamine unit of the chitosan. These interactions could lead to the conclusion that the glycerol not leached from the patch remains immobilized between the chitosan chains performing a secondary cross‐linking effect.

Further, a desirable wound dressing material should be permeable to water vapour to control the moisture of the wound, which is essential for the healing process. The ability of a patch to control water loss can be determined by the WVTR, which is the steady state at which water vapour permeates the dressing at specified conditions of temperature and relative humidity. In particular, an extremely high value of WVTR may lead to the dehydration of the wound, self‐defeating the purpose of wound healing: in fact, a moist environment has several benefits on cell communication and migration, which result in a faster and more efficient healing process. On the contrary, an excessively low WVTR value can cause the accumulation of wound exudates, which can lead to bacteria accumulation and wound infection, and the dilution of active molecules locally delivered on the wound site.^[^ ^70^, ^71^ ^]^

The WVTR for normal skin is 204 g m^−2^ day^−1^, while for wounded skin it can range between 279 g m^−2^ day^−1^ to 5138 g m^−2^ day^−1^), for first‐degree burn and granulating wound, respectively. Therefore, the WVTR for materials with wound dressing application should be 250–2500 g m^−2^ day^−1^.^[^ ^9^, ^72^ ^]^

From the data reported in Figure 3c, the WVTR of the examined SL‐patches falls in the middle of the suggested values. In general, the water vapour permeability of a polymeric patch mainly depends on the presence of pores, its degree of hydrophilicity, the presence and state of dispersion of solid fillers therein, the amount of the polymer crystalline phase, and its molecular mobility. Due to the presence of a wide range of hydrophilic groups in the examined samples, a great value of vapour permeability was expected^[^ ^60^ ^]^ and confirmed by the results obtained for all the SL‐patches, which exhibited WVTR values ranging between 1136 and 1241 g m^−2^ day^−1^ on day 1. However, from the data reported in the literature,^[^ ^73^ ^]^ a decrease in the WVTR value of the patches was expected, with the increasing amount of tannic acid. Anyway, no correlation between the WVTR values and the amount of tannic acid was detected in the analysed patches. This was probably due to the minimal percentage of tannic acid employed for the SL‐patches and the water‐vapour transmission rate ability of the patch is mainly driven by the other factors described above. Finally, we repeated the tests at different time points (1, 3, and 7 days) to evaluate if the vapour transmission ability changes with moisture absorption. The WVTR for all the samples remained stable through all time points, proving that the water vapour permeability of the patch is not influenced by the exposure time to a humid environment.

To collect further evidence on the chemistry of patches, the pure substances (chitosan, glycerol and tannic acid) and the obtained SL‐patches were analysed by FTIR‐ATR, however no evident differences were observed form the three spectra which result as the sum of all typical peaks of chitosan and glycerol and due to their prevalence in the composition of membranes. Instead, no signals of tannic acid were observed. (Figure S2a, Supporting Information).

### 3.3 Mechanical Properties

**Figure 4:**
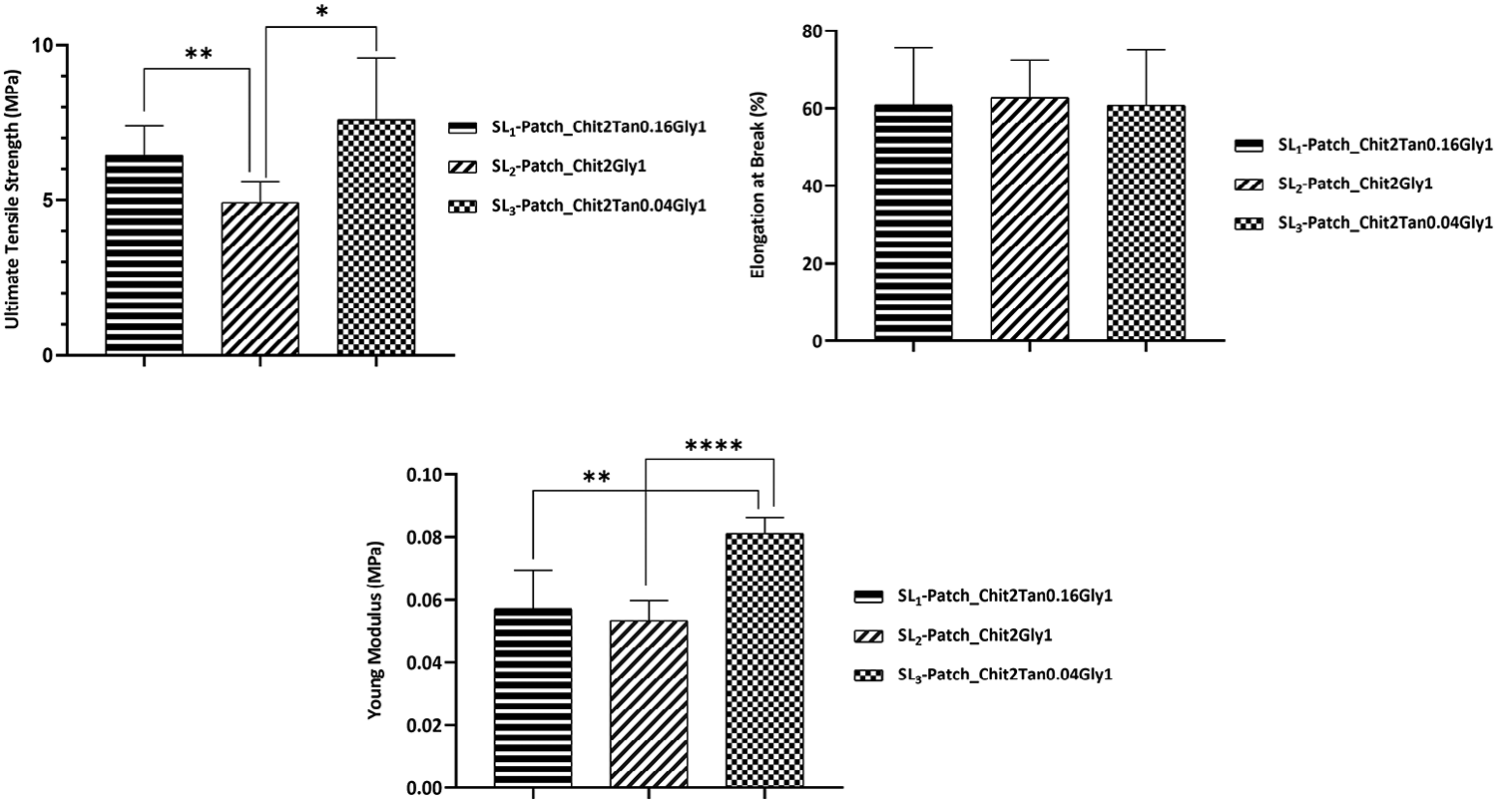
Mechanical properties of the SL‐patches: Ultimate Tensile Strength, Elongation at Break, Young Modulus. One‐Way ANOVA followed by Un‐paired *t*‐test. * *p *≤ 0.05, ** *p *≤ 0.01, *** *p *≤ 0.005, **** *p *≤ 0.0001.

To be successfully employed as wound dressing, patches should have optimal mechanical properties to adapt to the body's movements while maintaining the integrity of the wound dressings. Ideally, they should have mechanical properties as close as possible to the ones of normal skin, which in literature is reported to have a tensile strength in the range of 2.5–16 MPa and an elongation at break of ≈70%.^[^ ^72^ ^]^ Here, the ultimate tensile strength (UTS), elongation at break (EB), and Young modulus (YM) of the SL‐patches were evaluated by tensile tests. The results obtained (**Figure** **4**) showed that all the patches exhibit the desired mechanical properties to be applied as a wound dressing. Specifically, the elongation at break for all the SL‐patches ranges between 60.8% and 62.7% with no significant difference between the patches’ composition, which could be attributed to the fact that glycerol employed as plasticizer is the component in the blend that mainly affects the EB, establishing hydrogen bonds with the chitosan chains and allowing greater movement between them. Therefore, the presence of glycerol reduces the stiffness of the membrane and increases its flexibility.^[^ ^20^ ^]^ Instead, tensile strength and Young modulus range respectively between 4.9–7.6 MPa, and 0.053–0.081 MPa, with significant differences depending on the composition of the SL‐patches. In particular, it was found that the addition of tannic acid in the blend exerts an effect on the ultimate tensile strength of the patch, with SL_2_‐patch (Chit2Gly1) exhibiting a significantly diminished UTS value (*P* < 0.05) if compared with tannic acid‐containing SL_1_ and SL_3_ patches. This effect can be attributed to the reinforcement of the cross‐linking throughout the chitosan matrix, independently from the amount of tannic acid which did not affect significantly (*P* > 0.05) the tensile strength of the patches. It resulted that SL_1_‐patch and SL_3_‐patch exhibited statistically comparable UTS values. This trend, however, is not confirmed by Young Modulus, for which the SL_3_‐patch displays a statistically and significantly higher value of YM (*P* < 0.005) compared to SL_1_‐patch, SL_2_‐patch. Therefore, a higher tensile stiffness for the patch containing the lower amount of tannic acid was observed, if compared to the patch containing a higher amount of cross‐linker, or no cross‐linker at all.

### 3.4 Antimicrobial Peptide LTX‐109 Loading on SL_2_‐Patch

Based on the performed tests, in particular water uptake and in vitro degradability, SL_2_‐patch (Chit2Gly1) was chosen, among the other SL‐patches prepared, as the best candidate for antimicrobial peptide loading, as it has exhibited a higher swelling degree and degradation rate, which are both factors able to promote a good interchange with the wound site, thus resulting in an effective controllable drug release. To prepare the loaded patch (SL_2_‐patch‐L), the antimicrobial peptide LTX‐109 was pre‐solubilized in ethanol, and then it was added to the blend and stirred to obtain a homogeneous solution. A predefined volume was cast in a Petri dish and a thin medicated patch was obtained through solvent evaporation. The amount of AMP selected for this application was established considering the antimicrobial activity of LTX‐109 and thus achieving the MIC in the eluates used later for the evaluation of drug activity and preservation.^[^ ^74^ ^]^ Furthermore, it has been defined to avoid a too high concentration in the wound site which could be responsible for the creation of a toxic microenvironment dangerous not only for pathogens but also for cell proliferation and therefore unfavourable for wound healing processes.

**Figure 5:**
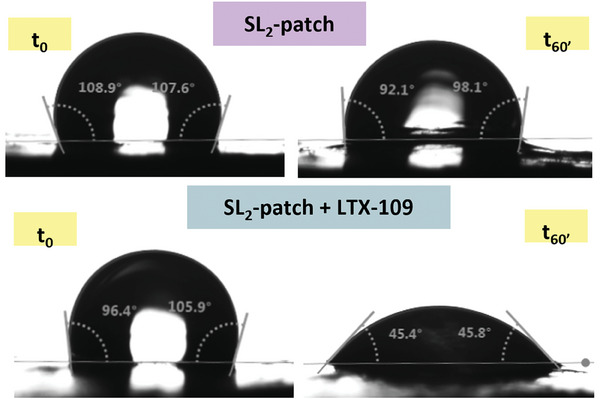
Contact angles measurements for SL_2_‐patch (Chit2Gly1) and SL_2_‐patch‐L (Chit2Gly1+LTX).

Evidence for the incorporation of the AMP into the patch was collected through contact angle measurements.^[^ ^75^ ^]^ The average contact angles of the SL_2_‐patch without and with the incorporation of the LTX‐109 were 107.44 ± 1.11 and 105.65 ± 6.38 at the initial time t_0_, respectively (**Figure** **5**). After one minute (*t*_60_) they decreased to 95.30 ± 0.30 and 47.13 ± 2.20, respectively. The significant change in the contact angle observed for the LTX‐109‐loaded patch is due to an increase in hydrophilicity following the incorporation of the peptide, due to a modification of the patch surface through both chemical and physical interaction of the antimicrobial peptide with the material matrix, in the form of hydrogen bonds between the amine and carbonyl groups of the peptide and the amine and alcohol groups of the elements comprising the SL_2_‐patch matrix (chitosan and glycerol), and physical entrapment of the peptide itself in the porosity of the patch.

Further, were collected FTIR‐ATR graphs of pure LTX‐109 and of SL_2_‐patch and SL_2_‐patch‐L, however, the only peaks clearly visible from the spectra of patches are those of chitosan and glycerol as they are the two main components of the membranes (Figures S2b, Supporting Information).

### 3.5 LTX‐109 Release Profile from SL_2_‐Patch‐L

**Figure 6:**
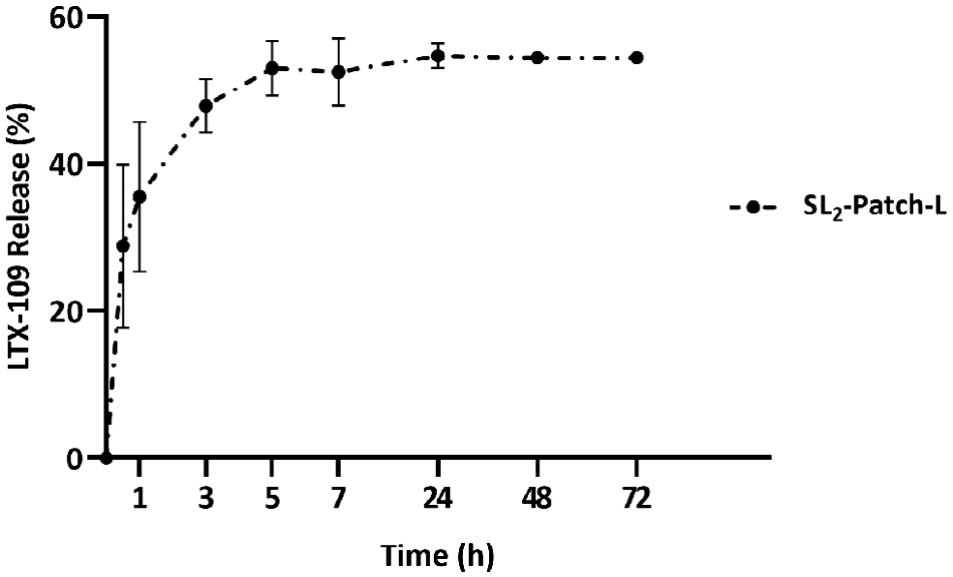
LTX‐109 kinetic release profile from SL_2_‐patch‐L in PBS at 37 °C.

The kinetic release of LTX‐109 from the SL_2_‐patch‐L (Chit2Gly1+LTX‐109) was evaluated in phosphate buffer saline solution (PBS, pH = 7.2, 37 °C) by measuring its concentration with the high‐performance liquid chromatography (HPLC). The retention time of the LTX‐109 released from the patch perfectly matches that registered from the standard solution used for the calibration curve, assessing the preservation of the molecule (Figure S4, Supporting Information). The collected profile (**Figure** **6**) revealed a burst release of the AMP, as more than the 40% was released from the patch during the first hour. Then, the release steadily continued until 72 h, reaching a plateau at the 55% of the total content, a release profile closely resembling patterns described in the literature for other devices reliant on diffusion‐controlled release mechanism, where the drug to be released is uniformly distributed throughout a polymer matrix and exposed on the surface. In these cases, the device often initially exhibits a burst release followed by a rapidly declining drug release rate.^[^ ^76^ ^]^ This outcome underscores the potential efficacy of the medicated SL_2_‐patch‐L for effectively treating and hopefully controlling infections in the wound site,^[^ ^28^ ^]^ thanks to the early and fast release of the antimicrobial agent and its prolonged permanence inside the patch, which could prevent the proliferation of bacteria and the onset of secondary infections.

However, this test was performed in PBS, a much more neutral environment compared to the highly proteolytic environment of wound exudates. In these conditions, the material likely exhibits a higher degradation, with the possible release of all the AMP amount into the wound site within the first 24h and without a sustained drug release in the following hours. The initial burst release, while serving well in managing the acute phase of the infection, promptly suppressing bacterial colonisation and offering immediate relief to the patients, unfortunately offer limited protection to the antimicrobial peptide against degradation caused by protease within wounds, leading to its metabolization without being effectively utilised. On the other hand, a sustained release of AMPs from the patch can play a pivotal role in the chronic wound care treatment. By ensuring a prolonged, consistent wound disinfection, it aptly supports the extended healing process of chronic wounds, minimizes the need for frequent reapplications and reduces patient discomfort. Furthermore, the controlled release strategy establishes a protective shield, isolating the residual drug within the patch from direct protease contact. This barrier effectively curtails the risk of premature degradation, ensuring the AMPs remain functional and potent throughout the treatment course.^[^ ^28^, ^76^, ^77^ ^]^

### 3.6 Multi‐Layer Design and Characterization

**Figure 7:**
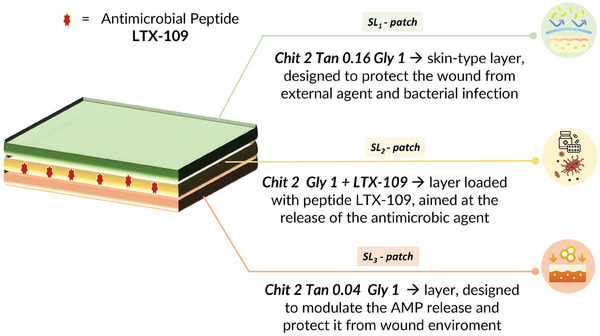
Schematic representation of the developed multi‐layer patch structure.

To obtain a device that allowes a controlled and prolonged AMP release over time and ensures a greater antimicrobial durability, the SL‐patches described above were combined in a three‐layered structure (ML‐patch) as schematized in **Figure** **7**. In particular, the designed structure was composed by:

An upper skin‐like layer made of the SL_1_‐patch (Chit2Tan0.16Gly1). It exhibited a lower swelling degree and degradation rate, and a good water vapor permeability, which makes it suitable for lasting protection of the disrupted skin from external agents.

A middle layer made of the SL_2_‐patch (Chit2Gly1), which is suitable for the effective release of the antimicrobial peptide, as described before. The protection within SL_1_ and SL_3_ further guarantees its stability in the wound microenvironment and allows prolongation and further control of the AMP release over time.

A bottom layer made of SL_3_‐patch Chit2Tan0.04Gly1, characterized by a lower crosslinking degree compared with the top layer and enhanced swelling abilities. It can regulate the release of AMP in the wound site, protect it from the highly aggressive environment to preserve its function, and, thanks to the healing properties of its constituent materials (chitosan and tannic acid) it has the potential to assist the regeneration of the disrupted skin.

This complex design and the permeability of SL_1_ and SL_3_ will guarantee further to exert bacterial protection in both, the upper (against external contamination) and the bottom (directly on wound) directions.

### 3.7 ML‐Patch Morphological Characterization

The multi‐layered patch was obtained through solvent‐casting technique, with a pre‐established volume of each blend solution that was cast in a Petri dish and allowed to dry at 40 °C until a firm but still adhesive surface was obtained before casting the following layer. This procedure guarantees good adhesion within the stacked films and avoids the diffusion of AMP in both external layers. Then, the finished patch was obtained, with a thickness of 0.4 mm, through the completion of the solvent evaporation at room temperature.

**Figure 8:**
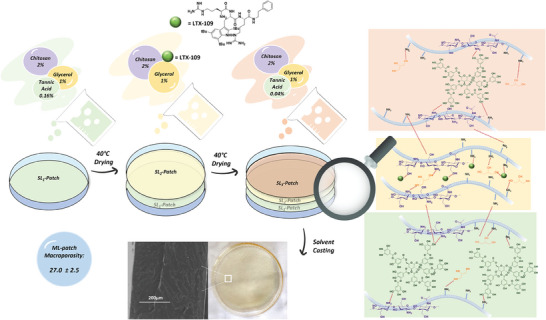
Scheme of the preparation sequence to obtain the ML‐patch, ESEM morphology of its cross‐section, and ML‐patch macro‐porosity value.

The morphology of the ML‐patch was evaluated macroscopically by appearing as a uniform, flat, and semi‐transparent film similar to SL‐patches, and microscopically by analysing the cross‐section of the sample with ESEM. As shown in **Figure** **8**, the multi‐layer exhibited a uniform and compact structure, and no clear separation between the layers was observed, which indicates the achievement of good adhesion between them. The macroporosity found for the ML‐patch is 27%, a slightly higher value if compared to the ones of the SL‐patches reported above.

### 3.8 ML‐Patch Physical Characterization

**Figure 9:**
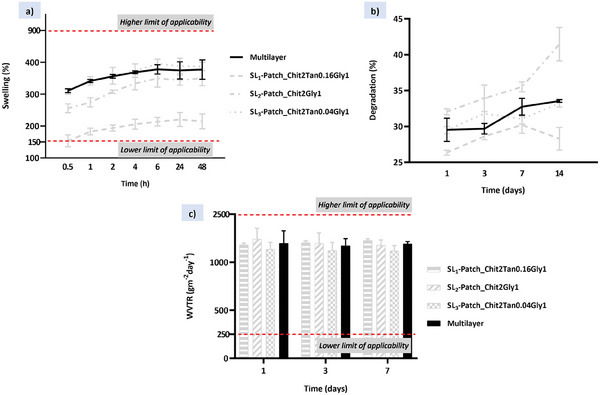
Physical properties of the ML‐patches: a) water uptake percentages of ML‐patch in PBS within 48 h; b) weight loss percentages of ML‐patch in PBS within 7 days; c) water vapor transmission rate of the ML‐patch at 1, 3, and 7 days. The data collected for the ML‐patch are compared with the values collected from the SL‐patches, reported in light‐grey lines.

The water uptake ability of the ML‐patch was also evaluated (**Figure** **9**a) to predict the performance of the patch in absorbing the exudates in the wound site. After six hours the ML‐patch reaches its maximum swelling value, ≈370% of its dry weight, which is within the range defined by the highest and the lowest swelling value found for the SL‐patches (respectively SL_1_ and SL_3_). This indicates that the water uptake ability of the ML‐patch is in line with the average swelling value of those of the SL‐patches that compose it. Similarly, in vitro degradation was also evaluated in PBS at 37 °C and the results were comparable with the results obtained for the SL‐patches (Figure 9b). There was an initial weight loss of ≈30%, mostly due to the glycerol solubilization, and then there were no significant changes in the degradation values after 14 days in PBS, which indicated the long‐term stability of the patches. Finally, the multi‐layer water vapor permeability was measured and resulted in 1920 g m^−2^ day^−1^, a value comparable with the ones found for the SL‐patches evaluated above (Figure 9c).

### 3.9 ML‐Patch Mechanical Properties

**Figure 10:**
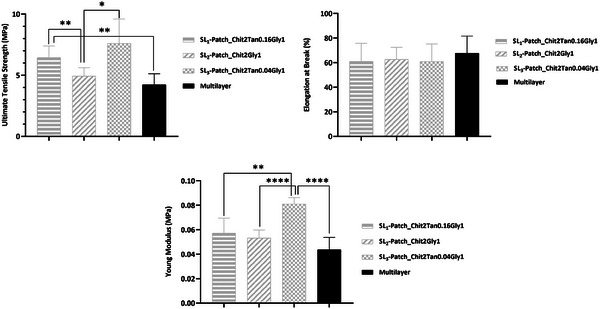
Mechanical properties of the ML‐patch: Ultimate Tensile Strength, Elongation at Break, Young Modulus. The data collected for the ML‐patch are compared with the values collected from the SL‐patches, reported in light‐grey lines. One‐Way ANOVA followed by Un‐paired *t‐*test. * *p *≤ 0.05, ** *p *≤ 0.01, *** *p *≤ 0.005, **** *p *≤ 0.0001.

The mechanical properties of the ML‐patch were evaluated, and the results are summarized in **Figure** **10**. This material presents elongation at break (EB) of 67.7%, ultimate tensile strength (UTS) of 4.2 MPa, and Young Modulus (YM) of 0.049 MPa. The values are in agreement with the desired requirements for material for wound dressing applications. Moreover, the ML‐patch did not present a significant difference in EB value from those of the SL‐patches reported above (*P* > 0.05). On the contrary, UTS and YM significantly differ from SL‐patch ones. Namely, the UTS of ML‐patch exhibits a dissimilar behavior (*P *< 0.005) if compared to SL_1_‐patch and SL_3_‐patch, cross‐linked with tannic acid. Then, its UTS is close to the ones of the not cross‐linked SL_2_‐patch, which can be correlated with the layered structure of the patch that decreases its resistance to breakage. As for Young Modulus, ML‐patch exhibits a significantly different behavior (*P* < 0.005) from SL_3_‐patch, and comparable (*P* > 0.05) to less stiff materials like SL_1_‐patch and SL_2_‐patch. The lower mechanical performances highlighted for the ML‐patch, in particular, compared to those of the SL_3_‐patch, can be explained by considering the thickness of every single layer in the ML device is lower than that of the SL_3_‐patch. Indeed, the SL_3_‐patch has a thickness of 0.2 mm, while in the ML‐patch, the total thickness is 0.4 mm whereas the upper layer (SL_3_ composition) is only 0.13 mm thick.

### 3.10 Antimicrobial Peptide Loading on ML‐Patch

To finely tune the release of the antimicrobial peptide LTX‐109 from the device when applied to the wound, the peptide was incorporated in the middle layer of the ML‐patch. LTX‐109 was first solubilized in ethanol and added to the Chit2Gly1 solution, then the multi‐layer assembling was performed as described above to obtain the medicated ML‐patch‐L.

The effect of the correct incorporation of peptide LTX‐109 in the multi‐layer was examined through contact angle measurements (Figure S3, Supporting Information). In this case, as the peptide was added only in the middle layer, no significant changes in the contact angle of the top (SL_1_‐patch) and the bottom (SL_3_‐patch) layer were expected as a result of LTX‐109 loading. In accordance with this hypothesis, the average contact angle values of the top layer of the ML‐patch before and after the peptide incorporation were 107.95 ± 5.98 and 98.11 ± 2.69 at *t*_0_, and 77.33 ± 6.49 and 74.88 ± 3.48 at *t*_60_. Similarly, the average contact angle values of the bottom layer without and with the peptide incorporation were 100.80 ± 1.84 and 103.48 ± 4.47 at *t*_0_, and 73.58 ± 2.18 and 75.11 ± 7.08 at *t*_60_ (Figure S3, Supporting Information). Since the examined contact angle values do not change with the peptide incorporation, it is possible to conclude that the LTX‐109 was effectively incorporated only in the middle layer of the ML‐patch, without leaking in the top or bottom layers.

### 3.11 LTX‐109 Release Profile from ML‐Patch‐L

**Figure 11:**
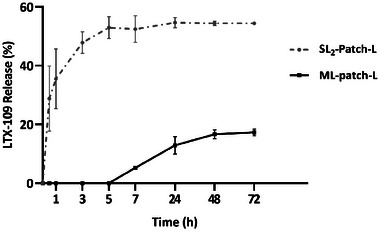
LTX‐109 release profile from ML‐patch‐L (black) and Chit2Gly1 SL_2_‐patch‐L (grey).

As described in Section 3.5, the release of antimicrobial peptide LTX‐109 from the ML‐patch‐L was evaluated in phosphate buffer saline solution (PBS, pH = 7.2, 37 °C) by HPLC monitoring. The release profile is reported in **Figure** **11**, contextually to the already discussed LTX‐109 release profile from the SL_2_‐patch‐L. It is possible to observe a considerably slower release of the antimicrobial peptide LTX‐109 from the ML‐patch‐L, indicating that the multi‐layer patch offers greater resistance to drug diffusion in the surrounding medium. In particular, the burst release observed for the SL_2_‐patch‐L was not detected for the ML‐patch‐L, as it started after 5 h and substantially increased during days 1 to 3, reaching 18% of peptide release at day 3 (72 h). This observation aligns with the fact that within the ML‐patch‐L the antimicrobial peptide LTX‐109 is not homogeneously distributed across the material's surface. Instead, its loading is concentrated exclusively in the middle layer, away from the surface, meaning that the AMP must undergo diffusion to the adjacent layers before being eventually released in the surrounding environment, which is a favorable feature both because it places a physical barrier between the peptide and the proteases present at the site of infection, and because it ensures a slower release.^[^ ^76^ ^]^

Despite the arguably low amount of peptide released during the first hours, which however can be easily modulated by modifying the initial concentration of LTX‐109 reached in the polymer blend before drying, the more controlled release confirms our hypothesis about the advantages of a three‐layered structure allowing finer modulation of the release profile and thus of the local drug administration.

Specifically, with this approach, the material can be personalized and suitably tuned for applications in highly proteolytic environments such as infected and chronic wounds, where it can ensure a local and gradual drug release and therefore a sustained antimicrobial activity without the need for remove the dressing patch, helping to reduce the incidence of cytotoxicity in the adjoining tissues and the risk of protease induced peptide degradation. The residual LTX‐109 (45% for SL_2_‐patch‐L and 82% for ML‐patch‐L (Figure 11), remaining embedded into the polymeric matrix, continues to exert a prolonged antimicrobial activity during and until complete degradation of the patch.

### 3.12 Antibacterial and Cytotoxicity Assay

The antibacterial activity of the fabricated medicated patches (SL_2_‐patch‐L, and ML‐patch‐L) was assessed in vitro by measuring the inhibitory effect of the antimicrobial peptide LTX‐109 released into PBS solutions following different incubation intervals. This experimental setting was chosen to evaluate the effectiveness of the AMP‐loaded patches over time in a model system mimicking the physiological conditions.

In a preliminary set of experiments, the reference strains, *S. aureus* and *P. aeruginosa*, were assayed with the PBS solutions, and related serial dilutions, in which the unloaded SL_2_‐ and ML‐patches were incubated for 1, 24, and 48 h. Bacterial proliferation was not affected by these samples, thus indicating that reagents used for patch preparation did not interfere with bacterial viability and that all inhibitory properties measured by testing AMP‐loaded patches are related to the LTX‐109 synthetic peptide.

**Figure 12:**
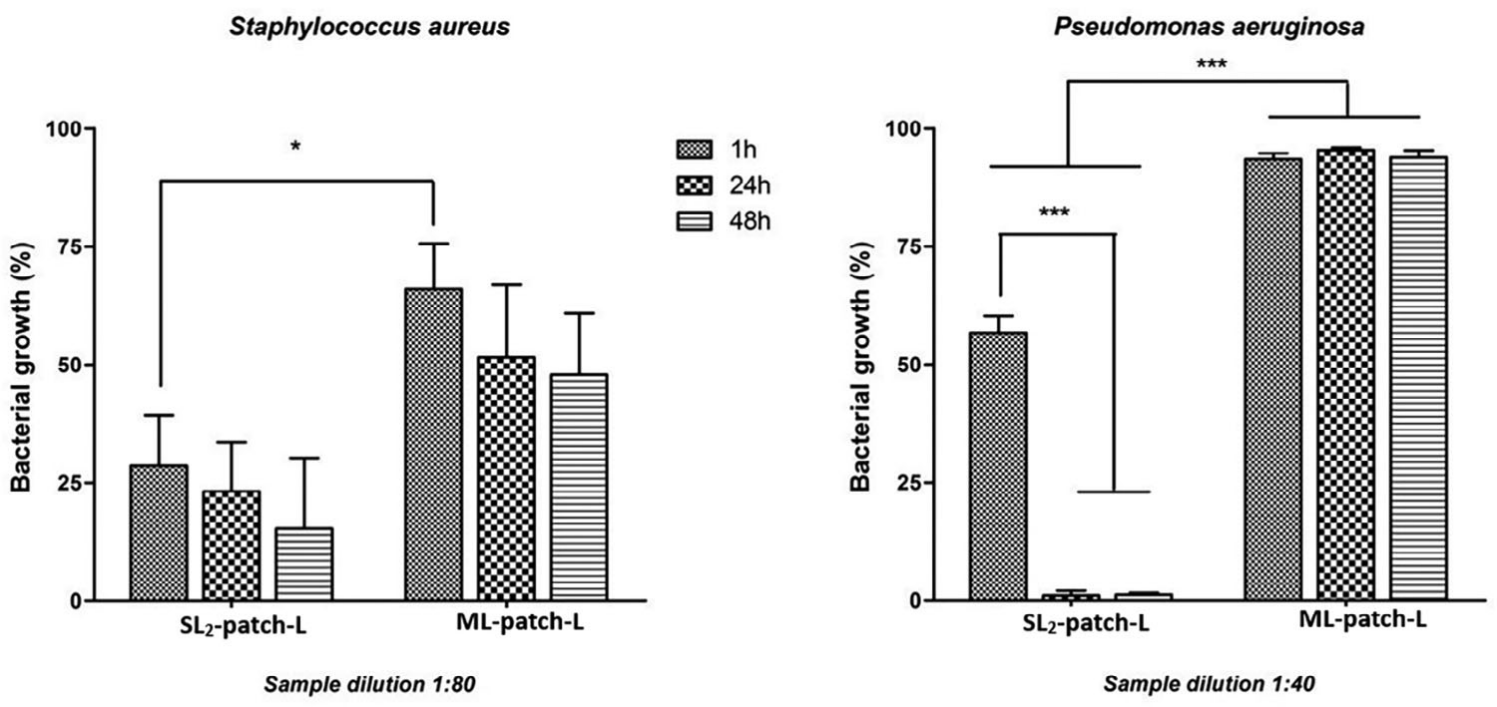
Antibacterial activity of the LTX‐109 released in PBS solutions, at different time intervals, from (SL_2_‐patch‐L, and ML‐patch‐L). Data are expressed as percentage values of the bacterial growth relative to the positive control. Error bars represent standard deviation. One‐Way ANOVA and unpaired *t*‐tests were carried out. * *p *< 0.05, * *p *< 0.0001.

As for the AMP‐loaded patches both mono‐ and multi‐layered samples (SL_2_‐patch‐L, and ML‐patch‐L), displayed inhibitory activity on bacterial growth with different effectiveness depending on the tested biomaterials and bacterial strains. **Figure** **12** details the bacterial proliferation of the reference strains assayed with selected dilutions of PBS samples (dilution 1:80 for *S. aureus* and dilution 1:40 for *P. aeruginosa*). Both SL_2_‐patch‐L and ML‐patch‐L displayed remarkable inhibitory activity toward the tested strains with a higher effect on Gram‐positive compared to Gram‐negative. This finding can be ascribed to the lower permeability of *P. aeruginosa* compared to *S. aureus*, due to the presence of the outer membrane and lipopolysaccharides in the Gram‐negative cells. These structures increase the permeability threshold of Gram‐negative strains to many active molecules and many classes of clinically effective Gram‐positive antibiotics.^[^ ^78^, ^79^ ^]^

Regardless of the dilution assayed and the tested strains, the inhibitory effect of the antimicrobial peptide released by SL_2_‐ and ML‐patches‐L increased as a function of the incubation time, reducing bacterial growth, perfectly matching with the previously reported LTX‐109 kinetic profiles. As for samples obtained from SL_2_‐patch‐L, LTX‐109 contained in PBS solutions after 1 h of incubation drastically reduced bacterial growth by 71.4% and the following samplings displayed an increasingly high inhibitory effect up to 84.7%. Notably, for SL_2_‐ ML‐patches‐L on *S. aureus* and SL_2_‐patch‐L on P. aeruginosa, 48 h samples maintained the same activity as the related samples collected at 24 h, thus indicating a long‐term inhibitory potential.

**Figure 13:**
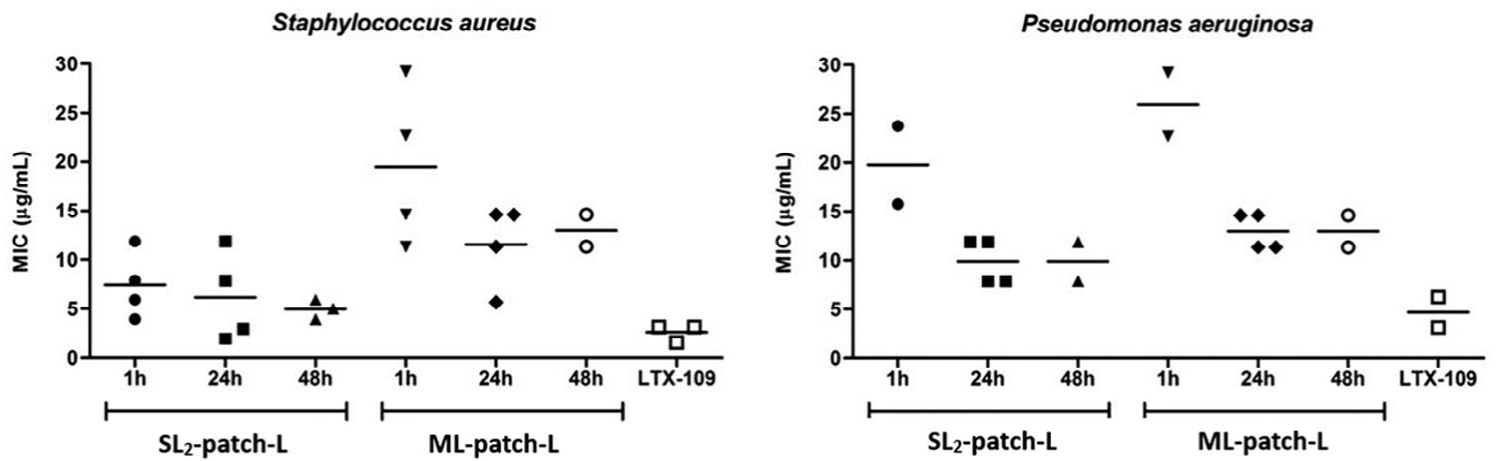
MIC values obtained for the LTX‐109 released in PBS solutions from SL_2_‐ and ML‐patches‐L, and for the synthetic peptide. The scatter dot plot reports MIC values measured for different experiments and the mean values.

The inhibitory property of the LTX‐109 released in the liquid solutions by the different patches was also evaluated by considering the amount of peptide incorporated during the synthesis of the layers. Taking into account the dilutions tested in the broth microdilution assays, MIC values were determined for both patches on *S. aureus* and *P. aeruginosa*, and the synthetic peptide as a reference sample (Figure 13, Table S2, Supporting Information). Results confirm the high inhibitory activity of samples indicating that the prepared patches made of chitosan, plasticized with glycerol, and cross‐linked with tannic acid are suitable to preserve the potency of the synthetic peptide LTX‐109. As all samples (1, 24, and 48 h) completely inhibit the bacterial growth at a defined concentration leading to the determination of MIC values, it is possible to affirm that all biomimetic AMP‐loaded patches release the proper amount of active LTX‐109 that guarantee the inhibition of bacterial proliferation. Moreover, MIC values measured for the LTX‐109 peptide (reference sample) were very close to those obtained for liquid solutions when patches were incubated for 24 and 48 h, thus confirming the aptness of the used biomaterials. As for the LTX‐109 peptide, MIC values measured on *P. aeruginosa* were slightly higher than those obtained on *S. aureus*, as a result of the lower activity of the peptide itself on Gram‐negative bacteria, thus not related to its loading of the patches.

The biological relevance of data obtained on the reference bacterial strains was further evaluated in light of experiments performed on mammalian cells to assess the cytotoxicity of the devised biomaterials. For these assays, the samples, previously tested for their inhibitory activity on bacterial growth, were analyzed for their effects on Hel 299 cells after 48 h of treatment. This cell line was selected as a model system of no‐malignant human fibroblasts. None of the samples obtained from AMP‐loaded and unloaded SL_2_‐patches, at the different time intervals and the highest inhibitory dilution, affected cell proliferation, thus confirming their cytocompatibility (Figure S5, Supporting Information).

**Figure 14:**
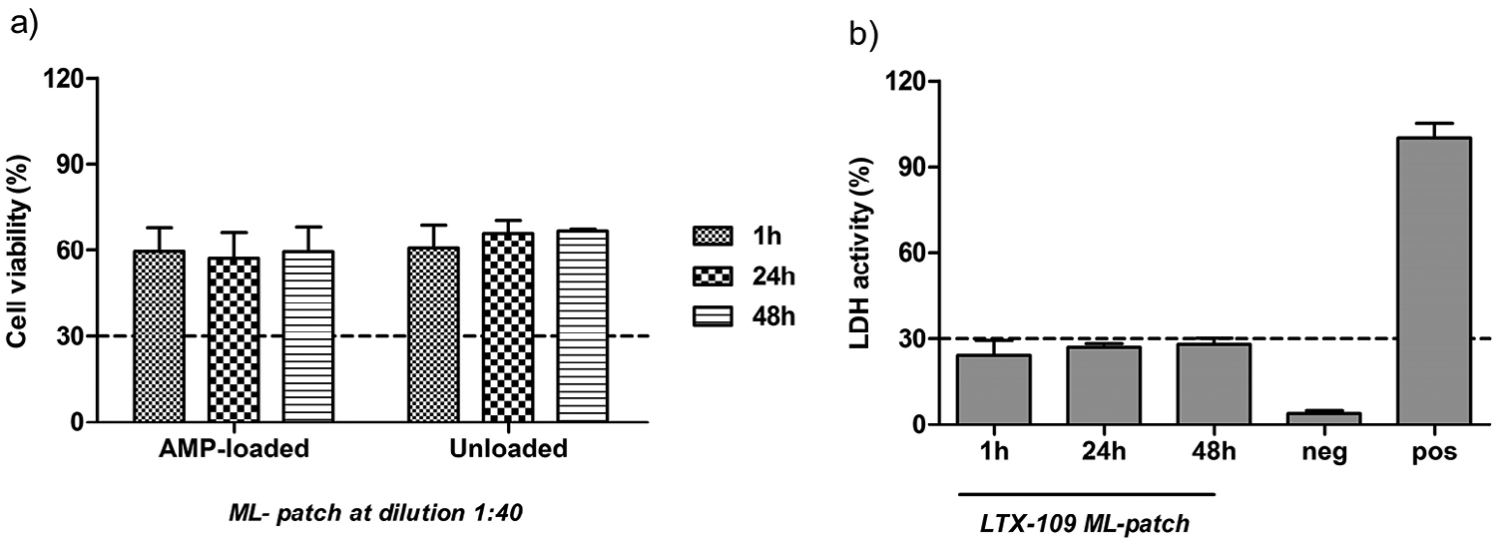
Cell viability a) and LDH activity b) measured in Hel 299 incubated for 48 h with PBS solutions, at different time intervals, obtained from ML‐patches at the highest antibacterial dilution (1:40). Data are expressed as percentage values relative to the untreated cells. Error bars represent standard deviation. The horizontal line is set at 30%.

As for ML‐patches, Hel 299 viability decreased compared to untreated cells following the incubation with PBS samples collected at 1, 24, and 48 h, at the highest bacterial inhibitory dilution, regardless of the loading with LTX‐109 (**Figure** **14**a). The overall viability of cells treated with the AMP‐loaded samples was 58.7 ± 13.1% while for the unloaded samples was 64.5 ± 8.5%. No differences were measured between the two categories of samples; these results perfectly match the complete safety profile of the LTX‐109 peptide when tested in the range of 25–0.19 µg mL^−1^ (Figure S6, Supporting Information). Given these observations, the reduced viability of Hel 299 cells incubated with ML‐patches could be related to a higher amount of glycerol released in the cell media, due to the higher weight of the ML‐patch compared to the SL_2_‐patch, rather than the loaded antimicrobial peptide.

To delve into this result, the culture media recovered from mammalian cells treated with the liquid solutions and relative to the multi‐layered AMP‐loaded patch were tested for cytotoxicity. The amount of LDH enzyme, which is a measure of damaged membranes, was measured at the different experimental conditions, and cell death was expressed as relative to untreated cells and reference lysed samples (100% of lysis) (Figure 14b). The average value of cytotoxicity for ML‐patches was 29.05 ± 6.6% which is an acceptable level of biocompatibility for medical device materials (<30% according to ISO10993‐5).

## 4 Conclusion

In this work, a multi‐layered patch for the treatment of chronic wounds has been designed looking in the direction of the transition from simple dressing to healing through an approach of prolonged local disinfection and stimulation of tissue regeneration. It was designed to i) protect the wound from external contamination; ii) provide long‐term local diffusion of a selective drug; iii) control bacterial growth; iv) avoid frequent medication that interferes with the natural wound healing process; v) support the cellular mechanism responsible for skin tissue regeneration.

Three single layers, mainly composed of chitosan, plasticized with glycerol and cross‐linked with tannic acid, were successfully prepared by solvent casting method and medicated by the loading of the antimicrobial peptide LTX‐109, selected for its rapid and broad‐spectrum action. Each of them has been thought for a specific function and differs in terms of chemical compositions and degree of cross‐linking.

Every single layer has been fully characterized and arranged accordingly to its properties for the obtainment of a stable and multilayer patch that demonstrated excellent properties in terms of water‐vapour transmission rate, water uptake capability, and degradability, thus meeting the necessary requirements to be applied as dressing in chronic wounds. Furthermore, the incorporation of the LTX‐109 in the middle layer guarantees a sustainable and slow release of the antimicrobial peptide; the LTX‐109 was efficiently released from the multi‐layered patch over a period of 48 h and its potency was fully preserved as demonstrated by MIC values obtained on the tested reference bacteria. Indeed, the loaded biomaterials yield the proper amount of active LTX‐109 that guarantees the inhibition of bacterial proliferation. Finally, in vitro assays performed on non‐malignant mammalian cells indicate a safety profile for all devised AMP‐loaded patches.

In summary, the results collected with this study confirm that our design is functional to the development of an effective device able of performing multiple functions and transferring multiple signals in contact with the wound environment. The upper, more external skin‐like layer has been demonstrated to be durable and suitable for protective functions, the medicated intermediate layer has allowed the local, controlled, and long‐lasting release of the antimicrobial peptide and the lower layer has been demonstrated to be suitable to perform a regenerative function proving its ability to maintain the required environmental conditions in terms of moisture, disinfection, and breathability.

## Conflict of Interest

The authors declare no conflict of interest.

## Author Contributions

S.B. and L.F. contributed equally to this work. Conceptualization was dealt by S.M., C.E., B.S., F.L., and T.A. Methodology was dealt by B.S., C.E., F.L., and P.C. Validation was done by B.S., A.C., B.F., G.G.A., F.L., and P.C. Formal Analysis was done by B.S., A.C., B.F., P.C., and F.L. Resources were dealt by T.A. and S.M. Data Curation was done by B.S., A.C., B.F., and F.L. B.S., C.E., F.L., and B.F. was dealt with writing the Original draft. Writing the review and editing was done by S.M. and T.A., and G.G.A. Supervision was done by S.M. Project administration was done by S.M. and T.A. Funding acquisition was done by T.A. and S.M. All authors have read and agreed to the published version of the manuscript.

## Supporting information



Supporting Information

Supplemental Video 1

## Data Availability

The data that support the findings of this study are available from the corresponding author upon reasonable request.

## References

[1] P. Deng , W. Jin , Z. Liu , M. Gao , J. Zhou , Carbohydr. Polym. 2021, 260, 117767. 33712125 10.1016/j.carbpol.2021.117767

[2] S. Torkaman , H. Rahmani , A. Ashori , S. H. M. Najafi , Carbohydr. Polym. 2021, 258. 10.1016/j.carbpol.2021.117675 33593551

[3] Y. He , W. Zhao , Z. Dong , Y. Ji , M. Li , Y. Hao , D. Zhang , C. Yuan , J. Deng , P. Zhao , Q. Zhou , Int. J. Biol. Macromol. 2021, 167, 182. 33259842 10.1016/j.ijbiomac.2020.11.168

[4] T. R. Johnson , B. I. Gómez , M. K. McIntyre , M. A. Dubick , R. J. Christy , S. E. Nicholson , D. M. Burmeister , Int. J. Mol. Sci. 2018, 19, 2699. 30208569 10.3390/ijms19092699 PMC6164292

[5] S. R. Nussbaum , M. J. Carter , C. E. Fife , J. DaVanzo , R. Haught , M. Nusgart , D. Cartwright , Value Health 2018, 21, 27. 29304937 10.1016/j.jval.2017.07.007

[6] C. K. Sen , Adv. Wound Care 2021, 10, 281. 10.1089/wound.2021.0026 PMC8024242 33733885

[7] S. Dhivya , V. V. Padma , E. Santhini , BioMedicine 2015, 5, 24. 26615539 10.7603/s40681-015-0022-9 PMC4662938

[8] A. Moeini , P. Pedram , P. Makvandi , M. Malinconico , G. Gomez d'Ayala , Carbohydr. Polym. 2020, 223, 115839. 10.1016/j.carbpol.2020.115839 32059889

[9] H. Mndlovu , L. C. du Toit , P. Kumar , Y. E. Choonara , T. Marimuthu , P. P. D. Kondiah , V. Pillay , Macromol. Chem. 2020, 25, 222. 10.3390/molecules25010222 PMC6982769 31935794

[10] Z. Li , Z. Lin , Aggregate. 2021, 2, e21.

[11] G. Suarato , R. Bertorelli , A. Athanassiou , Front. Bioeng. Biotechnol. 2018, 6, 137. 30333972 10.3389/fbioe.2018.00137 PMC6176001

[12] J. Qin , F. Chen , P. Wu , G. Sun , Front. Bioeng. Biotechnol. 2022, 10, 841583. 35299645 10.3389/fbioe.2022.841583 PMC8921732

[13] D. N. Moholkar , P. S. Sadalage , D. Peixoto , A. C. Paiva‐Santos , K. D. Pawar , Eur. Polym. J. 2021, 160, 110784.

[14] Y. Yao , A. Zhang , C. Yuan , X. Chen , Y.a Liu , Biomater. Sci. 2021, 9, 4523. 34047308 10.1039/d1bm00411e

[15] A. O. Ijaola , D. O. Akamo , F. Damiri , C. J. Akisin , E. A. Bamidele , E. G. Ajiboye , M. Berrada , V. O. Onyenokwe , S. Y. Yang , E. Asmatulu , J. Biomater. Sci., Polym. Ed. 2022, 33, 1998. 35695023 10.1080/09205063.2022.2088528

[16] X. Deng , M. Gould , M. A. Ali , J. Biomed. Mater. Res., Part B 2022, 110, 2542. 10.1002/jbm.b.35086 PMC9544096 35579269

[17] Y. Liang , Y. Liang , H. Zhang , B. Guo , Asian J Pharm. Sci. 2022, 17, 353. 35782328 10.1016/j.ajps.2022.01.001 PMC9237601

[18] C. Shi , C. Wang , H.e Liu , Q. Li , R. Li , Y. Zhang , Y. Liu , Y. Shao , J. Wang , Front. Bioeng. Biotechnol. 2020, 8, 182. 32266224 10.3389/fbioe.2020.00182 PMC7096556

[19] J. Y. Hao , F. L. Mi , S. S. Shyu , Y. B. Wu , J. Y. Schoung , Y. H. Tsai , Y. Bin Huang , J. Biomed. Mater. Res. 2002, 59, 438. 11774301 10.1002/jbm.1260

[20] M. S. Pacheco , G. E. Kano , L. D.e A. Paulo , P. S. Lopes , M. A. de Moraes , Int. J. Biol. Macromol. 2020, 152, 803. 32068057 10.1016/j.ijbiomac.2020.02.140

[21] M. Mirhaj , S. Labbaf , M. Tavakoli , A. M. Seifalian , Int. Wound J. 2022, 19, 1934. 35297170 10.1111/iwj.13786 PMC9615294

[22] L. Heras , M. Igartua , E. Santos‐Vizcaino , R. M. Hernandez , J. Controlled Release 2020, 328, 532. 10.1016/j.jconrel.2020.09.039 32971198

[23] M. Soliman , A. A. Sadek , H. N. Abdelhamid , K. Hussein , Res. Vet. Sci. 2021, 137, 262. 34052571 10.1016/j.rvsc.2021.05.013

[24] A. C. Nilsson , H. Janson , H. Wold , A. Fugelli , K. Andersson , C. Håkangård , P. Olsson , W. M. Olsen , Antimicrob. Agents Chemother. 2015, 59, 45. 10.1128/AAC.03513-14 PMC4291342 25331699

[25] J. Isaksson , B. O. Brandsdal , M. Engqvist , G. E. Flaten , J. S. M. Svendsen , W. Stensen , J. Med. Chem. 2011, 54, 5786. 21732630 10.1021/jm200450h

[26] B. E. Haug , W. Stensen , M. Kalaaji , Ø. Rekdal , J. S. Svendsen , J. Med. Chem. 2008, 51, 4306. 18570363 10.1021/jm701600a

[27] J. Håkansson , J. P. Cavanagh , W. Stensen , B. Mortensen , J.‐S. Svendsen , J. Svenson , J. Antibiot. 2021, 74, 5. 10.1038/s41429-021-00406-5 33495549

[28] A. P. Gomes , J. F. Mano , J. A. Queiroz , I. C. Gouveia , Carbohydr. Polym. 2015, 127, 451. 25965504 10.1016/j.carbpol.2015.03.089

[29] N. Rezaei , H. G. Hamidabadi , S. Khosravimelal , M. Zahiri , Z. A. Ahovan , M. N. Bojnordi , B. S. Eftekhari , A. Hashemi , F. Ganji , S. Darabi , M. Gholipourmalekabadi , Int. J. Biol. Macromol. 2020, 164, 855. 32640321 10.1016/j.ijbiomac.2020.07.011

[30] R. K. Thapa , D. B. Diep , H. H. Tønnesen , Acta Biomater. 2020,103, 62. 10.1016/j.actbio.2019.12.025 31874224

[31] M. L. Mangoni , A. M. Mcdermott , M. Zasloff , Exp. Dermatol. 2016, 25, 167. 26738772 10.1111/exd.12929 PMC4789108

[32] F. Miao , Y. Li , Z. Tai , Y. Zhang , Y. Gao , M. Hu , Q. Zhu , Macromol. Biosci. 2021, 21, 2100103. 10.1002/mabi.202100103 34405955

[33] F. Diban , S. Di Lodovico , P. Di Fermo , S. D'Ercole , S. D'Arcangelo , M. Di Giulio , L. Cellini , Int. J. Mol. Sci. 2023, 24, 1004. 36674518 10.3390/ijms24021004 PMC9862456

[34] H. Haidari , L. Melguizo‐Rodríguez , A. J. Cowin , Z. Kopecki , Am. J. Physiol.: Cell Physiol. 2023, 324, C29. 36409176 10.1152/ajpcell.00080.2022

[35] S. H. Jeong , S. Cheong , T. Y. Kim , H. Choi , S. K. Hahn , ACS Appl. Mater. Interfaces 2023, 15, 16471. 36943445 10.1021/acsami.3c00191

[36] S. Fahimirad , E. Ghaznavi‐Rad , H. Abtahi , N. Sarlak , Int. J. Pept. Res. Ther. 2021, 27, 2505.

[37] M. Albiero , A. Fullin , G. Villano , A. Biasiolo , S. Quarta , S. Bernardotto , C. Turato , M. Ruvoletto , G. P. Fadini , P. Pontisso , M. Morpurgo , Pharmaceutics 2022, 14, 1944. 36145692 10.3390/pharmaceutics14091944 PMC9503603

[38] J. A. Neff , D. F. Bayramov , E. A. Patel , J. Miao , Mil. Med. 2020, 185, 637. 32074338 10.1093/milmed/usz222 PMC7029774

[39] L. Yu , S. Dou , J. Ma , Q. Gong , M. Zhang , X. Zhang , M. Li , W. Zhang , Front. Mater. 2021, 8, 650223.

[40] H. Hamedi , S. Moradi , S. M. Hudson , A. E. Tonelli , Carbohydr. Polym. 2018, 199, 445. 30143150 10.1016/j.carbpol.2018.06.114

[41] J. Berger , M. Reist , J. M. Mayer , O. Felt , R. Gurny , Eur. J. Pharm. Biopharm. 2004, 57, 35. 14729079 10.1016/s0939-6411(03)00160-7

[42] P. Feng , Y. Luo , C. Ke , H. Qiu , W. Wang , Y. Zhu , R. Hou , L. Xu , S. Wu , Front. Bioeng. Biotechnol. 2021, 9, 650598. 33681176 10.3389/fbioe.2021.650598 PMC7931995

[43] N. Zoghi , M. H. Fouani , H. Bagheri , M. Nikkhah , N. Asadi , J. Appl. Polym. Sci. 2021, 138, 50781.

[44] R. Jayakumar , M. Prabaharan , P. T. Sudheesh Kumar , S. V. Nair , H. Tamura , Biotechnol. Adv. 2011, 29, 322. 21262336 10.1016/j.biotechadv.2011.01.005

[45] V. Patrulea , V. Ostafe , G. Borchard , O. Jordan , Eur. J. Pharm. Biopharm. 2015, 97, 417. 26614560 10.1016/j.ejpb.2015.08.004

[46] X. Ma , C. Qiao , X. Wang , J. Yao , J. Xu , Int. J. Biol. Macromol. 2019, 135, 240. 31128184 10.1016/j.ijbiomac.2019.05.158

[47] R. Shah , P. Stodulka , K. Skopalova , P. Saha , Polymers 2019, 11, 2094. 31847318 10.3390/polym11122094 PMC6960699

[48] A. Sionkowska , B. Kaczmarek , K. Lewandowska , J. Mol. Liq. 2014, 199, 318.

[49] N. Ninan , A. Forget , V. P. Shastri , N. H. Voelcker , A. Blencowe , ACS Appl. Mater. Interfaces 2016, 8, 28511. 27704757 10.1021/acsami.6b10491

[50] X. H. Shao , X. Yang , Y. Zhou , Q. C. Xia , Y. P. Lu , X. Yan , C. Chen , T. T. Zheng , L.‐L. Zhang , Y. u‐N. Ma , Y. u‐X. Ma , S. Z. Gao , Soft Matter 2022, 18, 2814. 35322837 10.1039/d2sm00058j

[51] H. Stenhamre , U. Nannmark , A. Lindahl , P. Gatenholm , M. Brittberg , J. Tissue Eng. Regen. Med. 2011, 5, 578. 21695799 10.1002/term.350

[52] E. Campodoni , E. B. Heggset , A. Rashad , G. B. Ramírez‐Rodríguez , K. Mustafa , K. Syverud , A. Tampieri , M. Sandri , Mater. Sci. Eng. C Mater. Biol. Appl. 2019, 94, 867. 30423774 10.1016/j.msec.2018.10.026

[53] M. K. Kumaran , J. Test Eval. 1998, 83.

[54] J. Xia , H. Zhang , F. Yu , Y. Pei , X. Luo , ACS Appl. Mater. Interfaces 2020, 12, 24370. 32368896 10.1021/acsami.0c05604

[55] R. Serra , R. Grande , L. Butrico , A. Rossi , U. F. Settimio , B. Caroleo , B. Amato , L. Gallelli , S. De Franciscis , Expert Rev. Anti Infect. Ther. 2015, 13, 605. 25746414 10.1586/14787210.2015.1023291

[56] A. Arora , A. Kothari , D. S. Katti , J. Mech. Behav. Biomed. Mater. 2015, 51, 169. 26256472 10.1016/j.jmbbm.2015.06.033

[57] P. Zahedi , I. Rezaeian , S. O. Ranaei‐Siadat , S. H. Jafari , P. Supaphol , Polym. Adv. Technol. 2010, 21, 77.

[58] S. Y. Ong , J. Wu , S. M. Moochhala , M. H. Tan , J. Lu , Biomaterials 2008, 29, 4323. 18708251 10.1016/j.biomaterials.2008.07.034

[59] V. Rubentheren , T. A. Ward , C. Y. Chee , C. K. Tang , Carbohydr. Polym. 2015, 115, 379. 25439908 10.1016/j.carbpol.2014.09.007

[60] P. Taheri , R. Jahanmardi , M. Koosha , S. Abdi , Int. J. Biol. Macromol. 2020, 154, 421. 32184139 10.1016/j.ijbiomac.2020.03.114

[61] F. Azadikhah , A. R. Karimi , G. H. Yousefi , M. Hadizadeh , Int. J. Biol. Macromol. 2021, 188, 114. 34358602 10.1016/j.ijbiomac.2021.08.006

[62] V. Acharya , A. Ghosh , A. R. Chowdhury , P. Datta , Soft Materials. 2021, 20, 149.

[63] Z. Bai , T. Wang , X. Zheng , Y. Huang , Y. Chen , W. Dan , Polym. Eng. Sci. 2021, 61, 278.

[64] A. Ulu , E. Birhanli , B. Ates , Int. J. Biol. Macromol. 2021, 188, 796. 10.1016/j.ijbiomac.2021.08.068 34400232

[65] J. Skrobot , W. Ignaczak , M. El Fray , Polym. Degrad. Stab. 2015, 120, 368.

[66] M. P. Sikka , V. K. Midha , Advanced Textiles for Wound Care. 2019, 463.

[67] S. Rivero , M. A. García , A. Pinotti , Carbohydr. Polym. 2010, 82, 270.

[68] I. Leceta , P. Guerrero , K. De La Caba , Carbohydr. Polym. 2013, 93, 339. 23465939 10.1016/j.carbpol.2012.04.031

[69] A. Domján , J. Bajdik , K. Pintye‐Hódi , Macromolecules 2009, 42, 4667.

[70] K. Nuutila , E. Eriksson , Adv. Wound Care 2021, 10, 685. 10.1089/wound.2020.1232 PMC8568799 32870777

[71] R. Xu , H. Xia , W. He , Z. Li , J. Zhao , B.o Liu , Y. Wang , Q. Lei , Y.i Kong , Y. Bai , Z. Yao , R. Yan , H. Li , R. Zhan , S. Yang , G. Luo , J. Wu , Sci. Rep. 2016, 6, 24596. 27086569 10.1038/srep24596 PMC4834567

[72] Y. E. Ma , L. Xin , H. Tan , M. Fan , J. Li , Y. Jia , Z. Ling , Y. Chen , X. Hu , Mater. Sci. Eng., C 2017, 81, 522. 10.1016/j.msec.2017.08.052 28888006

[73] A. L. A. Halim , A. Kamari , E. Phillip , Int. J. Biol. Macromol. 2018, 120, 1119. 30176328 10.1016/j.ijbiomac.2018.08.169

[74] L. D. Saravolatz , J. Pawlak , L. Johnson , H. Bonilla , L. D. Saravolatz , M. G. Fakih , A. Fugelli , W. M. Olsen , Antimicrob. Agents Chemother. 2012, 56, 4478. 22585222 10.1128/AAC.00194-12 PMC3421571

[75] X. Y. Zhang , C. Liu , P. S. Fan , X. H. Zhang , D. Y. Hou , J. Q. Wang , H. Yang , H. Wang , Z. Y. Qiao , J. Mater. Chem. B 2022, 10, 3624. 35420616 10.1039/d2tb00348a

[76] X. Huang , C. S. Brazel , J. Controlled Release 2001, 73, 121. 10.1016/s0168-3659(01)00248-6 11516493

[77] W. Wang , K. J. Lu , C. H. Yu , Q. L. Huang , Y. Z. Du , J. Nanobiotechnol. 2019, 17, 82. 10.1186/s12951-019-0514-y PMC6617859 31291960

[78] F. Bonvicini , I. Manet , F. Belluti , S. Gobbi , A. Rampa , G. A. Gentilomi , A. Bisi , ACS Infect. Dis. 2019, 5, 1524. 31264842 10.1021/acsinfecdis.9b00072

[79] H. Nikaido , Microbiol. Mol. Biol. Rev. 2003, 67, 593. 14665678 10.1128/MMBR.67.4.593-656.2003 PMC309051

