# Supplementary material for: Multiple‐Layer Chitosan‐Based Patches Medicated With LTX‐109 Antimicrobial Peptide for Modulated Local Therapy in the Management of Chronic Wounds
Source: Macromol Biosci. 2024 Oct 14;25(2):2400375. doi: 10.1002/mabi.202400375 (PMC11827553; doi:10.1002/mabi.202400375)
Supplement: Supplementary file 1 — Supporting Information [file MABI-25-2400375-s002.docx]

***Supplementary Information*
Multiple-layer chitosan-based patches medicated with LTX-109 antimicrobial peptide** **for modulated local therapy in the management of chronic wounds**

Sara Bernardoni^1†*^, Lucia Ferrazzano^2†^, Chiara Palladino^2^, Chiara Artusi^1^, Francesca Bonvicini^3^, Elisabetta Campodoni^1^, Giovanna Angela Gentilomi^3,4^, Alessandra Tolomelli^2^, Monica Sandri^1*^

**Table S1.** List of acronym frequently reported in the main text

| ACRONYM | DESCRIPTION |
| --- | --- |
| SL_1_-patch  (also called Chit2Tan0.16Gly1) | Single layer made of Chitosan (2%w/w_tot_) Tannic acid (0.16% w/w_tot_) Glycerol (1%w/w_tot_), with an higher degree of crosslinker (tannic acid) to act as the external, protective layer |
| SL_2_-patch  (also called Chit2Gly1) | Single layer made of Chitosan (2%w/w_tot_) and Glycerol (1%w/w_tot_), not crosslinked to guarantee the release of the antimicrobial peptide LTX-109 |
| SL_3_-patch  (also called Chit2Tan0.04Gly1) | Single layer made of Chitosan (2%w/w_tot_) Tannic acid (0.16% w/w_tot_) Glycerol (1%w/w_tot_), with an lower degree of crosslinker (tannic acid) with the function to regulate the LTX-109 release, and to provide regenerative stimuli |
| ML-patch | Multilayered, medicated patch composed by the SL-patch described above |
| SL_2_-patch-L | SL_2_-patch loaded with antimicrobial peptide LTX-109 |
| ML-patch-L | Multilayer patch loaded with antimicrobial peptide LTX-109 |
| AMP | Antimicrobial peptide, water-soluble host defence peptides found in various living organisms, that exhibit potent killing activity on a broad range of microorganisms with low tendency to induce resistance in vitro |
| LTX-109 | Novel broad-spectrum topical antimicrobial peptide (AMP) currently being investigated for the treatment of MRSA nasal decolonisation and bacterial skin infections. |
| PBS | Phosphate buffer saline (pH 7.2) |
| TFA | Trufluoroacetic acid |

a)


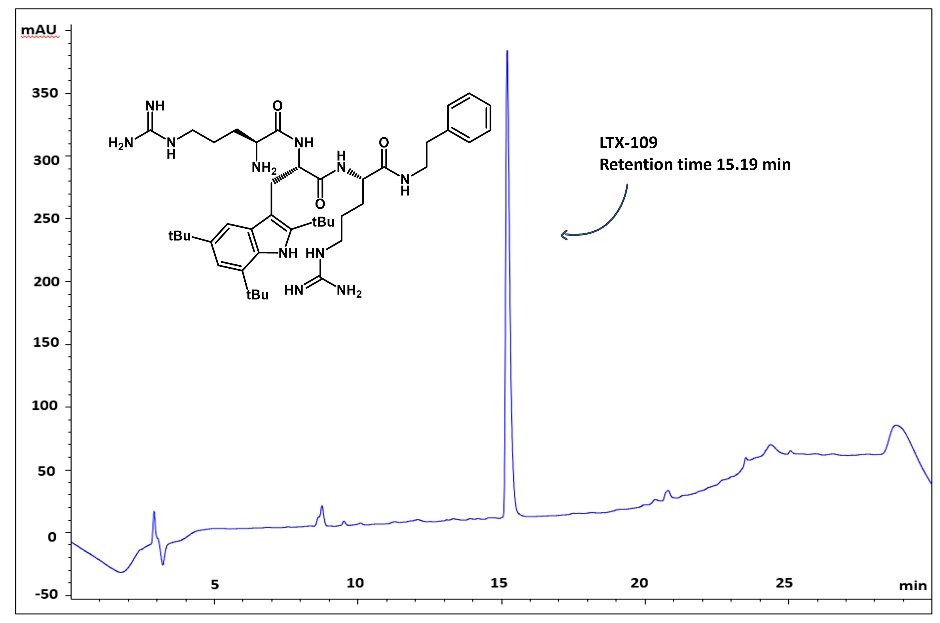


**b)** 1H-NMR δ(ppm): 7.41 (d, 1H, J=1.6 Hz), 7.28-7.08 (m, 6H), 4.60 (dd, 1H, J1=10.8 Hz, J2=4.4 Hz), 4.24 (m, 1H), 4.06 (m, 1H), 3.51 (dd, 1H, J1=14.8 Hz, J2=11.2 Hz), 3.28-2.94 (m, 7H), 2.55 (t, 2H, J=7.6 Hz), 2.04 (m, 2H), 1.73 (m, 3H), 1.53-1.28 (m, 3H), 1.49 (s, 9H), 1.44 (s, 9H), 1.39 (s, 9H).

**Figure S1. a)** Chromatogram of LTX-109, according to the analytical conditions reported in the main text; **b)** ^1^H-NMR signal map supporting the effectiveness and the purity of the synthesized LTX-109.


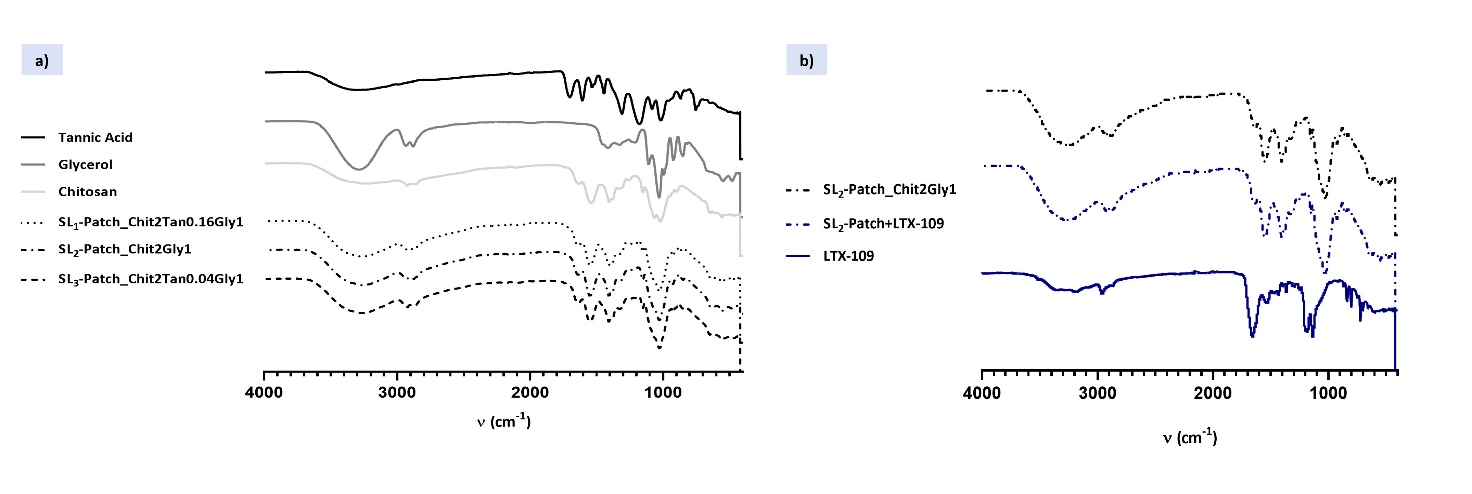


**Figure S2.** FTIR-ATR spectrograms of **a)** pure reactive compared with SL-patches; and **b)** of pure LTX-109 compared with SL_2_-patch loaded with LTX-109 and not loaded.


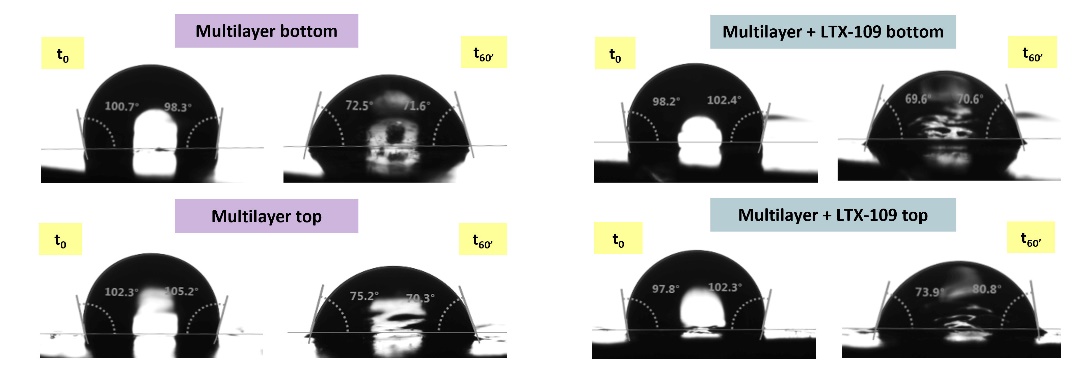


**Figure S3.** Contact angles measurements for multi-layer Top (composition of SL_1_-patch Chit2Tan0.16Gly1) and multi-layer Bottom (composition of SL_3_-patch Chit2Tan0.04Gly1) without and with the LTX-109.


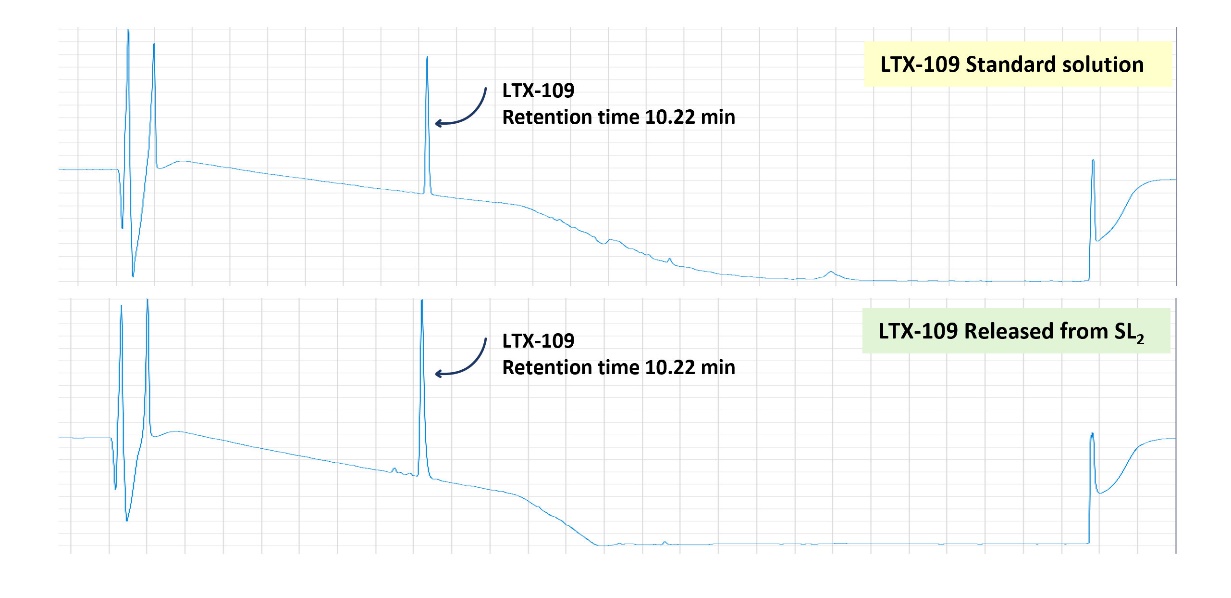


**Figure S4.** LTX-109 Chromatogram of pure LTX-109 standard (top) and LTX-109 released from the SL_2_-patch-L, according to the analytical conditions reported in the main text.

**Table S2.** MIC ranges (µg/mL) obtained on *S. aureus* ATCC 25293 and *P. aeruginosa* ATCC 27853 for the biomimetic AMP-loaded patches and the LTX-109 synthetic peptide.

| **Sample** | **S. aureus** | **P. aeruginosa** |
| --- | --- | --- |
| SL_2_-Patch-L 1h | 3.95-11.88 | 15.78-23.76 |
| SL_2_-Patch-L 24h | 1.97-11.88 | 7.89-11.88 |
| SL_2_-Patch-L 48h | 3.95-5.94 | 7.89-11.88 |
| ML-Patch-L 1h | 11.34-29.22 | 22.68-29.22 |
| ML-Patch-L 24h | 5.67-14.61 | 11.34-14.61 |
| ML-Patch-L 48h | 11.34-14.61 | 11.34-14.61 |


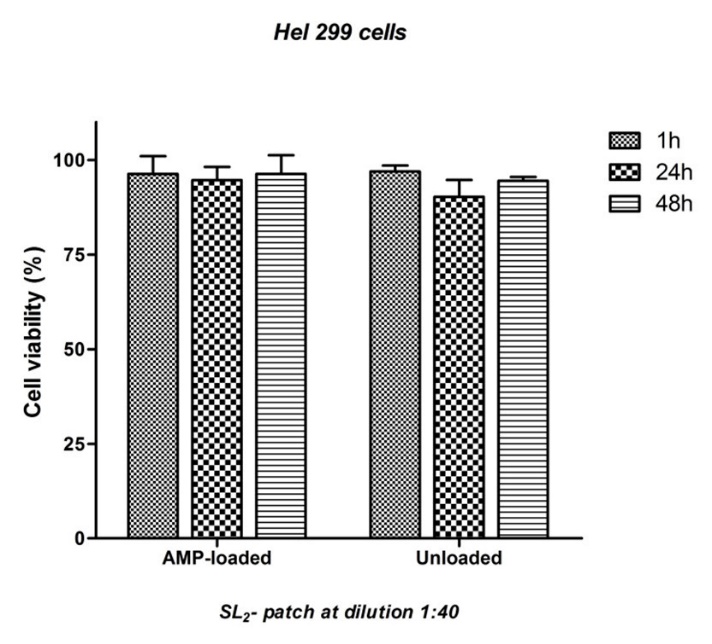


**Figure S5.** Cell viability and proliferation of Hel 299 incubated with PBS solutions, collected at different time intervals, obtained from SL_2_-patches-L and SL_2_-patches at the highest antibacterial dilution (1:40). Data are expressed as percentage values relative to the untreated cells. Error bars represent standard deviation.


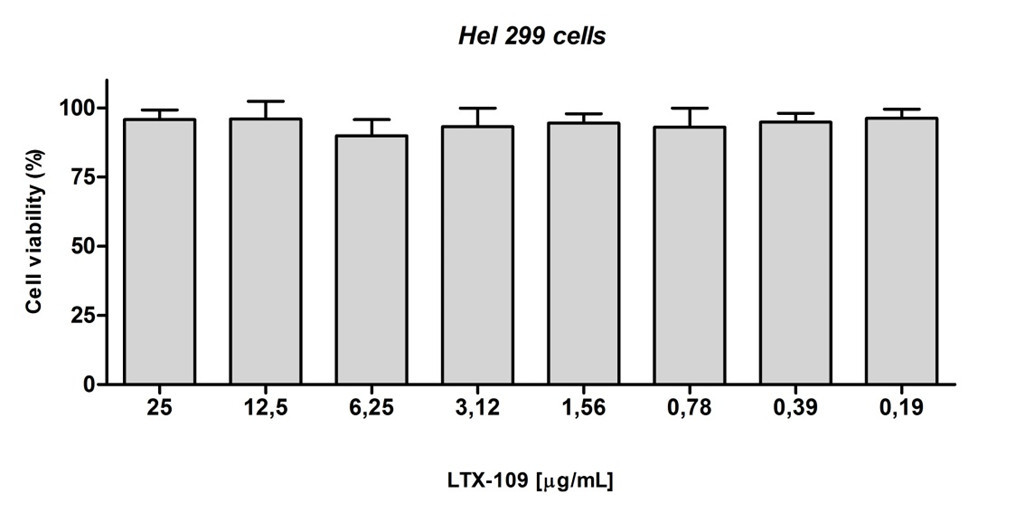


**Figure S6**. Cell viability and proliferation of Hel 299 treated for 48h with different concentrations of LTX-109 synthetic peptide. Data are expressed as percentage values relative to the untreated cells. Error bars represent standard deviation
